# Temozolomide promotes genomic and phenotypic changes in glioblastoma cells

**DOI:** 10.1186/s12935-016-0311-8

**Published:** 2016-05-05

**Authors:** Aleksei A. Stepanenko, Svitlana V. Andreieva, Kateryna V. Korets, Dmytro O. Mykytenko, Vladimir P. Baklaushev, Nataliya L. Huleyuk, Oksana A. Kovalova, Kateryna V. Kotsarenko, Vladimir P. Chekhonin, Yegor S. Vassetzky, Stanislav S. Avdieiev, Vladimir V. Dmitrenko

**Affiliations:** Department of Biosynthesis of Nucleic Acids, Institute of Molecular Biology and Genetics, National Academy of Science of Ukraine, Zabolotnogo str. 150, Kiev, 03680 Ukraine; Department of Medicinal Nanobiotechnology, Pirogov Russian State Medical University, Ostrovitianov str. 1, Moscow, 117997 Russia; Federal Research and Clinical Centre, FMBA of Russia, Orekhoviy Bulvar str. 28, Moscow, 115682 Russia; Department of Diagnostic of Hereditary Pathology, Institute of Hereditary Pathology, National Academy of Medical Sciences of Ukraine, Lysenko str. 31A, Lviv, 79008 Ukraine; Department of Experimental Cell System, R.E.Kavetsky Institute of Experimental Pathology, Oncology and Radiobiology, National Academy of Science of Ukraine, Vasylkivska str. 45, Kiev, 03022 Ukraine; Department of Human Genetics, Institute of Molecular Biology and Genetics, National Academy of Science of Ukraine, Zabolotnogo str. 150, Kiev, 03680 Ukraine; CNRS UMR8126, Institut de Cancérologie Gustave Roussy, Université Paris-Sud 11, Camille-Desmoulins str. 39, Villejuif, 94805 France

**Keywords:** Aneuploidy, Chromosome instability, Drug resistance, Glioma, Karyotype, Heterogeneity

## Abstract

**Background:**

Temozolomide (TMZ) is a first-line drug for the treatment of glioblastoma. Long-term TMZ-treated tumour cells acquire TMZ resistance by profound reprogramming of the transcriptome, proteome, kinome, metabolism, and demonstrate versatile and opposite changes in proliferation, invasion, in vivo growth, and drug cross-resistance. We hypothesized that chromosomal instability (CIN) may be implicated in the generation of TMZ-driven molecular and phenotype diversity. CIN refers to the rate (cell-to-cell variability) with which whole chromosomes or portions of chromosomes are gained or lost.

**Methods:**

The long-term TMZ-treated cell lines were established in vitro (U251TMZ1, U251TMZ2, T98GTMZ and C6TMZ) and in vivo (C6R2TMZ). A glioma model was achieved by the intracerebral stereotactic implantation of C6 cells into the striatum region of rats. Genomic and phenotypic changes were analyzed by conventional cytogenetics, array CGH, trypan blue exclusion assay, soft agar colony formation assay, scratch wound healing assay, transwell invasion assay, quantitative polymerase chain reaction, and Western blotting.

**Results:**

Long-term TMZ treatment increased CIN-mediated genomic diversity in U251TMZ1, U251TMZ2 and T98GTMZ cells but reduced it in C6TMZ and C6R2TMZ cells. U251TMZ1 and U251TMZ2 cell lines, established in parallel with a similar treatment procedure with the only difference in the duration of treatment, underwent individual phenotypic changes. U251TMZ1 had a reduced proliferation and invasion but increased migration, whereas U251TMZ2 had an enhanced proliferation and invasion but no changes in migration. U251TMZ1 and U251TMZ2 cells demonstrated individual patterns in expression/activation of signal transduction proteins (e.g., MDM2, p53, ERK, AKT, and ASK). C6TMZ and C6R2TMZ cells had lower proliferation, colony formation efficiency and migration, whereas T98GTMZ cells had increased colony formation efficiency without any changes in proliferation, migration, and invasion. TMZ-treated lines demonstrated a differential response to a reduction in glucose concentration and an increased resistance to TMZ re-challenge but not temsirolimus (mTOR inhibitor) or U0126 (MEK1/2 inhibitor) treatment.

**Conclusion:**

Long-term TMZ treatment selected resistant genotype-phenotype variants or generated novel versatile phenotypes by increasing CIN. An increase of resistance to TMZ re-challenge seems to be the only predictable trait intrinsic to all long-term TMZ-treated tumour cells. Changes in genomic diversity may be responsible for heterogeneous phenotypes of TMZ-treated cell lines.

**Electronic supplementary material:**

The online version of this article (doi:10.1186/s12935-016-0311-8) contains supplementary material, which is available to authorized users.

## Background

Temozolomide (TMZ), an imidazotetrazine derivative of the alkylating agent dacarbazine, is a first-line drug for the treatment of patients with glioblastoma. However, the TMZ efficiency is quite modest, with median overall survival ranging 9.4–19.0 months for radiotherapy combined with TMZ versus 7.3–17.1 months for radiotherapy alone [1]. TMZ is also used in the treatment of brain metastases, melanoma, lymphomas, refractory leukaemia, neuroendocrine tumours, pituitary tumours, Ewing’s sarcoma, primitive neuroectodermal tumours, lung cancer and other tumours [2]. Most tumour cells are intrinsically resistant or rapidly acquire resistance to TMZ at pharmacotherapeutic concentrations [3–6]. Long-term TMZ treatment of glioblastoma cells induced profound changes in heterochromatin organization and DNA methylation [7], transcriptome [8–12], proteome [13, 14], kinome [15], and metabolome [8, 10], remodeling of the entire electron transport chain and activation of oxidative stress responses [16, 17]. These changes impacted morphology, proliferation, adhesion, migration, invasion, and drug cross-resistance in a versatile manner [7, 8, 14, 18–23]. Such a complex phenotype adaptation certainly indicates intricate cellular and molecular defense mechanisms against TMZ. Additionally, the versatile phenotype responses to long-term TMZ treatment (Table 1) may point to the TMZ-promoted genome changes, which affect the organization and functionality of the genetic network (gene content, RNA and protein expression and their interaction). In fact, an acquisition of chemotherapy resistance is generally accompanied by genome evolution and, conversely, chromosomal instability (CIN) correlates with (multi)drug resistance [24–34].

**Table 1:** Long-term TMZ treatment of tumour cells results in versatile phenotype responses

Cell line	Morphology	Proliferation/viability	Cell cycle distribution	Migration/invasion	Growth in vivo/soft agar	Concentration/treatment period	Refs
A172	No change					100 μM/1 mo	[62]
C6	No change	↓		↓	↓ in soft agar	100 μM/1 mo in vitro or 50 mg/kg/10 injections in vivo	this study
D54	Changed	↓	↑ G0/G1 ↓ G2/M	↑		up to 0.5 mM/ 5 or 10 mo	[14]
CSC		↓			↓ in vivo		[10]
HEK293 derivatives	No change	↓			↓ in soft agar	up to 120 μM/ 3 mo	[23]
HeLa derivatives	No change	No change or ↑			↑ in soft agar	up to 120 μM/ 3 mo	[23]
Hs683		↓		↓	↓ in vivo	up to 1 mM/ 10 mo	[8]
LN-308, LNT-229, LN-18	No change	↓	No change			up to EC_50_/6 mo	[7]
T98G		↓		No change	No change in vivo	up to 1 mM	[19]
T98G	No change	No change		No change	↑ in soft agar	100 μM/1 mo	this study
U87	Changed			↑		up to IC_50_ = 150 µM/3 weeks	[18]
U251			↑ G2/M				[21]
U251	No change	↓ or ↑		↓ or ↑	No change in soft agar	up to 100 μM/ 5 or 10 weeks	this study
U373		↑		↑	↑ in vivo	up to 1 mM	[19]

CIN refers to the rate of gain or loss of whole chromosomes and portions of chromosomes, whereby the rate is defined as cell-to-cell variability or variability between cellular populations [35]. The dynamic numerical and structural chromosomal aberrations (genome chaos) result in profound alterations in gene expression, reprogramming of metabolic and signaling pathways and the generation of biochemical/phenotype diversification of cancer cells. Long-term drug-treated cells demonstrate transcriptomic and proteomic changes, and differ from parental cells at the molecular and cellular levels [26, 30]. Despite extensive studies, the role of CIN in the generation of TMZ-driven phenotype diversity and TMZ-based therapeutic failure has been poorly addressed.

Here, we characterized the genome-phenotype evolution of long-term TMZ-treated glioblastoma cell lines. TMZ treatment influenced genomic stability and phenotype diversity in a cell type-dependent manner by selecting resistant genotype-phenotype variants or generating novel versatile phenotypes by promoting CIN. Our data indicate that in addition to the reported TMZ-driven hypermutation phenotype [36–38], TMZ-instigated changes in genome stability and heterogeneity may contribute to the versatile phenotypic responses of tumour cells.

## Results

### Temozolomide promotes polyploidization and diverse karyotype changes

**Fig. 1:**
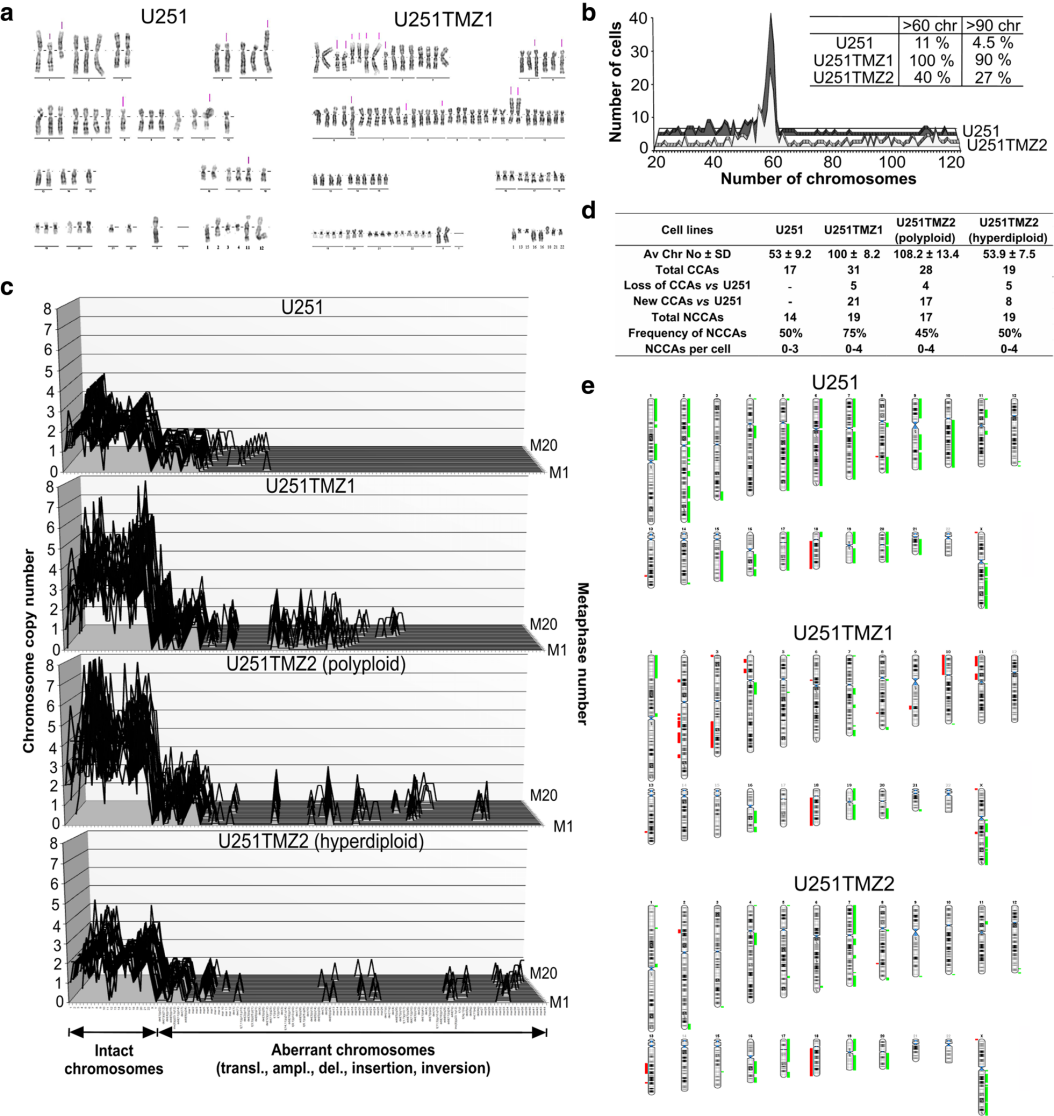
The TMZ-treated U251TMZ1 and U251TMZ2 have an increased CIN and ploidy. **a** Representative karyograms of U251 and U251TMZ1. The structurally abnormal chromosomes are marked. **b** Distribution of chromosomes across 200 metaphases of U251 and U251TMZ2 cell lines. The *insert table* shows a percentage of metaphases with numbers of chromosomes >60 or >90. **c** The karyotype differences between cell lines were demonstrated by alignment and comparison of karyographs of vehicle- and TMZ-treated derivatives. A list of all CCAs/NCCAs (in the same order as depicted on the *x*-*axis* of karyographs) and their copy number variation can be found in Additional file 1: Table S1. **d** A summary of karyotypic parameters of each cell line. **e** Chromosomal *ideograms* showing the areas of genetic gain/loss. *Bars* on the *left* (*red colour*) represent areas of copy number loss, whereas *bars* on the *right* (*green colour*) represent areas of copy number gain. Detailed description of copy number alterations (CNAs) can be found in Additional file 2: Table S2

To reveal the TMZ-promoted karyotypic and phenotypic changes, U251TMZ1, U251TMZ2, T98GTMZ, and C6TMZ cells were derived by repetitively exposing U251, T98G and C6 cells to TMZ (100 µM) in vitro, whereas C6R1 and C6R2TMZ cells were established in vivo [50 mg/kg, 10 intraperitoneal (i.p.) injections]. The vehicle-treated U251 cells were predominantly hyperdiploid with the mean number of chromosomes 53 ± 9.2; 11 % of cells contained more than 60 chromosomes/cell and 4.5 % of cells had more than 90 chromosomes/cell (calculated from 200 metaphases). In contrast, U251TMZ1 cells were mainly hypertetraploid with the mean number of chromosomes 100 ± 8.2 (90 %). The U251TMZ2 cells consisted of two subpopulations: a predominant subpopulation with a hyperdiploid karyotype (53.9 ± 7.5; 60 %) and a subpopulation with a hypertetraploid karyotype (108.2 ± 13.4; 27 %). A total portion of U251TMZ2 cells with more than 60 chromosomes/cell increased up to 40 % (Fig. 1a, b).

It was reported [11] that TMZ-resistant lines derived from a hyperdiploid SNB19 cell line (which is itself a derivative of U251 cells [23]) had deviations from a parental modal chromosome number or ploidy change, however, no analysis of CIN was performed. To visualize and compare CIN between cell lines, we used karyographs, 3-dimensional graphs, where x-axis designates the normal and aberrant chromosomes (clonal and non-clonal chromosome aberrations (CCAs/NCCAs), y-axis—the chromosome copy numbers, and z-axis—the numbers of metaphases arrayed for comparison to each other [33]. The karyographs show the degree of clonality and variability of chromosomes between individual cells of a cell line by comparing the copy numbers of intact and abnormal chromosomes of metaphases to each other. The karyotype differences between cell lines were demonstrated by alignment and comparison of karyographs of vehicle- and TMZ-treated derivatives. Karyotype changes of U251TMZ1 cells were accompanied by a loss of 5 CCAs, an acquisition of 21 new CCAs and a higher total number, frequency and per cell variation of NCCAs. Karyotype changes of U251TMZ2 hyperdiploid subpopulation were a loss of 5 and a gain of 8 new CCAs with an increase in the total number and per cell variation of NCCAs, whereas polyploid subpopulation was characterized by a loss of 4 and an acquisition of 17 new CCAs with an increase in the total number and per cell variation of NCCAs (Fig. 1c, d; Additional file 1: Table S1). Many CCAs were distinct between U251TMZ1 and U251TMZ2 cells. Analysis of array comparative genome hybridization (aCGH) data revealed striking differences in copy number alterations (CNAs) between U251, U251TMZ1 and U251TMZ2 cells (Fig. 1e; Additional file 2: Table S2).

**Fig. 2:**
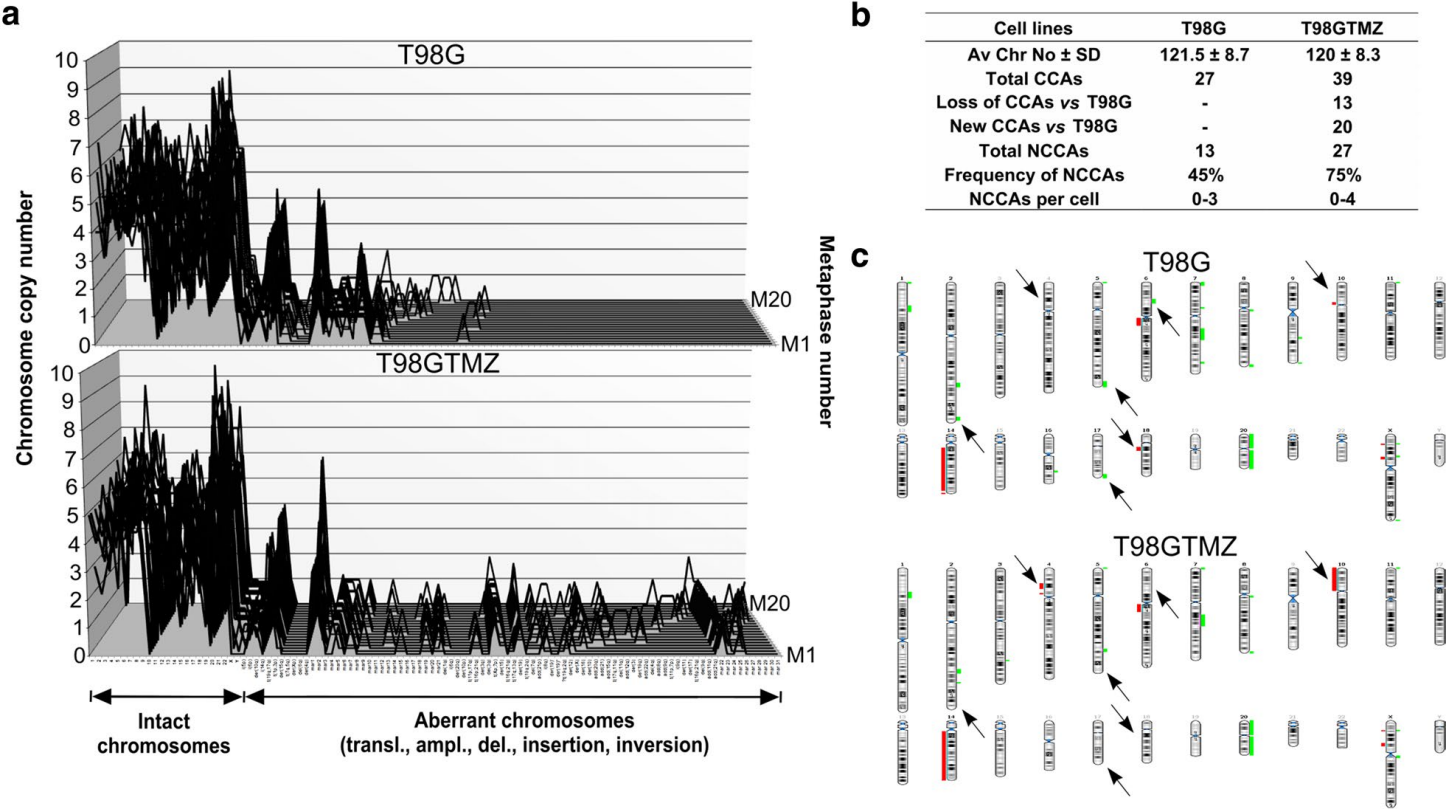
The TMZ-treated T98GTMZ have an increased CIN. **a** The karyotype differences between cell lines were demonstrated by alignment and comparison of karyographs of vehicle- and TMZ-treated derivatives. A list of all CCAs/NCCAs (in the same order as depicted on the *x*-*axis* of karyographs) and their copy number variation can be found in Additional file 3: Table S3. **b** A summary of karyotypic parameters of each cell line. **c** Chromosomal *ideograms* showing the areas of genetic gain/loss. *Bars* on the *left* (*red colour*) represent areas of copy number loss, whereas *bars* on the *right* (*green colour*) represent areas of copy number gain. Arrows depict the most obvious copy number alteration (CNA) differences between cell lines. Detailed description of copy number alterations (CNAs) can be found in Additional file 4: Table S4

Both T98G and T98GTMZ cells had a near-pentaploid karyotype with the mean numbers of chromosomes 121.5 ± 8.7 and 120 ± 8.3, respectively. Karyotype changes of T98GTMZ cells were accompanied by a loss of 13 CCAs, an acquisition of 20 new CCAs and a higher total number, frequency and per cell variation of NCCAs (Fig. 2a, b; Additional file 3: Table S3). The most obvious differences of CNAs between T98GTMZ and T98G cells were a loss of 4p15.2-p14 and 10p15.3-p11.21 in T98GTMZ cells and a gain of 2q37.1-q37.3, 5q35.1-q35.3, 6p22.1-p21.31, 17q25.1q25.3, and a loss of 18q11.2-q12.1 in T98G cells (Fig. 2c; Additional file 4: Table S4).

**Fig. 3:**
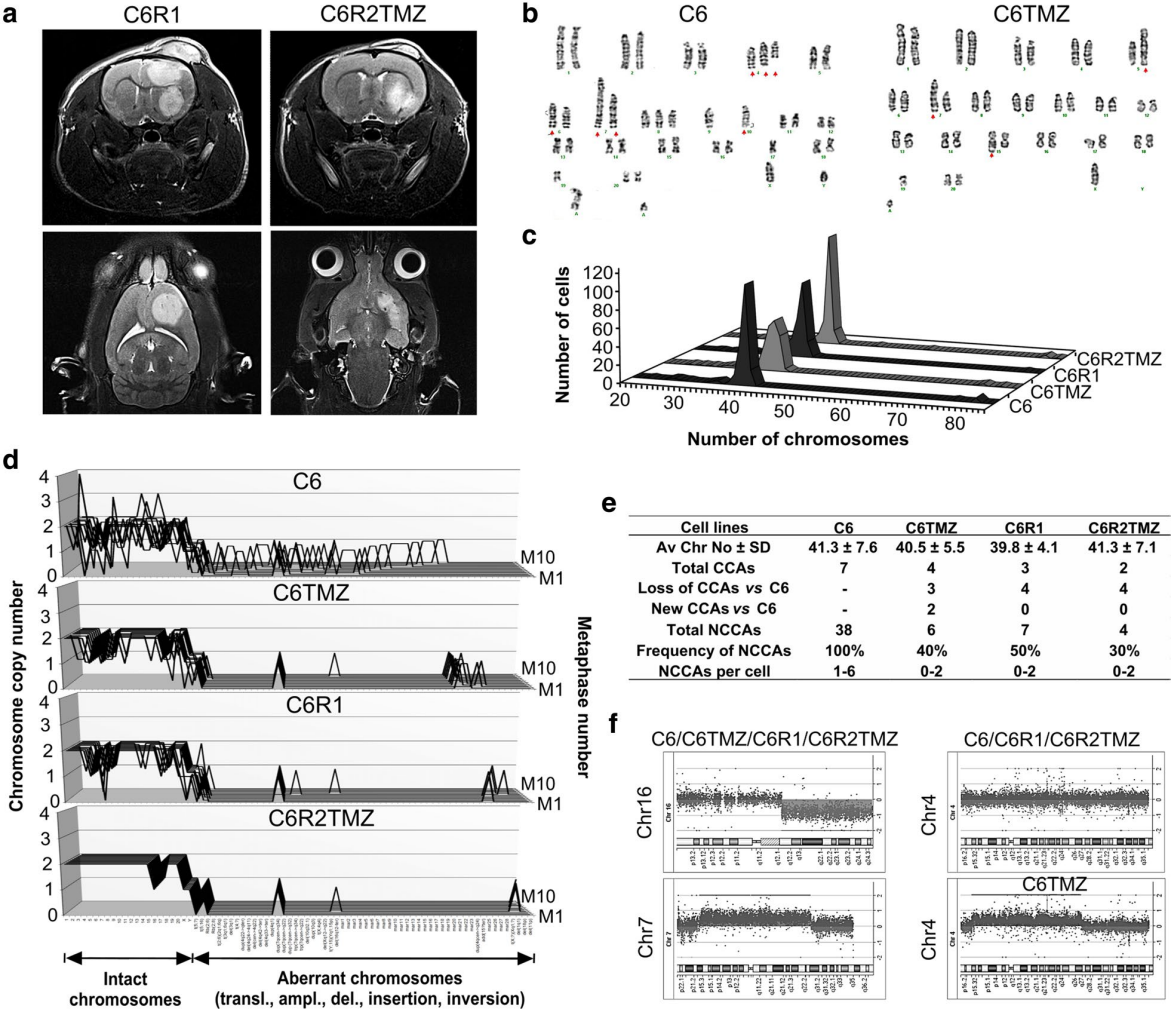
Characterization of the TMZ-treated C6TMZ, C6R2TMZ and the in vivo control C6R1 cell lines. **a** Brain magnetic resonance imaging (MRI) of the vehicle and TMZ-treated C6R1 and C6R2TMZ gliomas after 2 weeks of treatment. **b** Representative karyograms of C6 and C6TMZ. The structurally abnormal chromosomes are marked. **c** Distribution of chromosomes across 200 metaphases for each designated cell line. **d** The karyotype differences between cell lines were demonstrated by alignment and comparison of karyographs of vehicle- and TMZ-treated derivatives. A list of all CCAs/NCCAs (in the same order as depicted on the *x*-*axis* of karyographs) and their copy number variation can be found in Additional file 5: Table S5. **e** A summary of karyotypic parameters of each cell line. **f** The chromosome *ideograms* show the areas of genetic gain/loss. Detailed description of copy number alterations (CNAs) can be found in Additional file 6: Table S6

A morphometric analysis of C6R1 and C6R2TMZ glioma volume after 2 weeks of i.p. injection of DMSO or TMZ showed apparent differences in growth (≈75 versus ≈30 mm^3^) (Fig. 3a). C6TMZ, C6R1 and C6R2TMZ cells were near-diploid with the mean chromosome numbers 40–41 (±4.1–7.1) (Fig. 3b, c) and presented the selected subclones of karyotypically heterogeneous C6 cell line. Karyotype changes of C6TMZ cells and to a larger extent of C6R2TMZ cells were characterized by a reduction of a total number of CCAs and NCCAs. Interestingly, C6R1 also demonstrated a reduction of a total number of CCAs and NCCAs, suggesting that in vivo TMZ-treated cells underwent a two-stage selection: by the rat brain microenvironment and TMZ treatment (Fig. 3d, e; Additional file 5: Table S5). The major aberrations detected by aCGH and shared by C6 derivatives were a gain of 7p21.1-q31.1 and a loss of 16q12.1-q24.3. Additionally, C6TMZ but not C6R2TMZ cells demonstrated a gain of 4p16.1-q26 (Fig. 3f; Additional file 6: Table S6). Using the DAVID bioinformatics resource [39], a list of 613 well-annotated genes in this region was retrieved (Additional file 7: Table S7a). We also manually curated a list of published proteins/miRNAs that were shown to contribute to TMZ resistance (Additional file 8: Table S8). Then we cross-checked both lists and revealed at least 15 hits (marked in Additional file 7: Table S7a). Cross-checking with a list of 1221 putative genes extracted from the NCBI Map Viewer for the chromosomal region of interest produced several additional hits (marked in Additional file 7: Table S7B). Thus, TMZ treatment in vitro favoured selection of cells with a gain of the chromosomal region enriched in genes conferring resistance to TMZ. Copy number gain of the 4p16.1-q26 region in only in vitro TMZ-treated C6 cells may potentially result from a different TMZ concentration as well as in vitro versus in vivo cytotoxic effects. Firstly, tumour TMZ C_max_ varied at 20.6 ± 13.4 µM/L across glioma bearing rats after 20 mg/kg intra-venous (i.v.) injections [40]. Hence TMZ tumour concentration following 50 mg/kg i.p. injections, a dose used in this study, should still be lower than that used in culture (100 µM). Secondly, we and others demonstrated the formation in vivo of connexin 43-mediated gap junction channels between glioma cells and astroglia [41, 42]; this communication significantly reduces TMZ cytotoxicity [43].

### Temozolomide promotes versatile phenotype changes

**Fig. 4:**
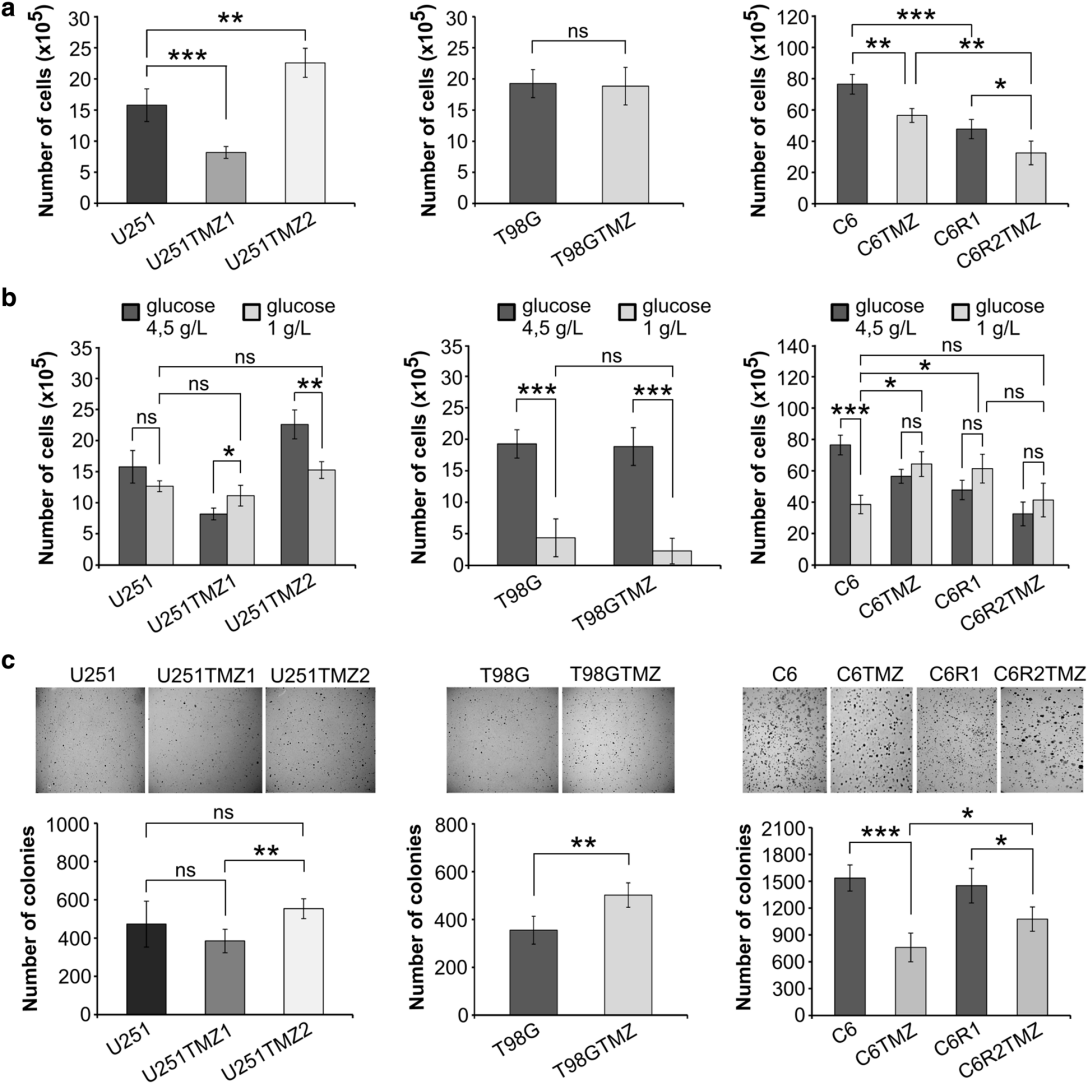
Long-term TMZ treatment promotes diverse changes in proliferation, sensitivity to a glucose concentration reduction and colony formation efficiency in soft agar. **a** Cell proliferation in high-glucose medium. Cells were seeded at a density of 5 × 10^4^ (U251 and T98G derivatives) or 1 × 10^4^ (C6 derivatives) and grown in a high-glucose (4.5 g/L) DMEM with 10 % FBS. On the 7th day of seeding, cells viability was evaluated by trypan blue exclusion assay. **b** Cell proliferation in high- glucose medium versus low-glucose medium (1 g/L). **c** Representative photographs of *plates* with *stained colonies* (*upper panel*) and graphs comparing colony formation efficiency of the designated cell lines (*lower panel*). *P < 0.05; **P < 0.01; ***P < 0.001; *NS* non significant

To elucidate how TMZ affected oncogenic characteristics of cells, we first analyzed cell proliferation. Previous studies demonstrated that the proliferation of long-term TMZ-treated glioblastoma cells was increased, decreased or unchanged (Table 1). U251 cells proliferated faster than U251TMZ1 cells but slower than U251TMZ2 cells. No difference in proliferation between T98G and T98GTMZ cells was observed. C6TMZ and C6R2TMZ cells proliferated slower than C6 and C6R1 cells, respectively. Furthermore, C6R1 and C6R2TMZ cells proliferated slower than C6 and C6TMZ cells, respectively (Fig. 4a), suggesting that the rat brain microenvironment might preferentially select for slower-dividing C6 cells. On the other hand, in vivo grown C6 derivatives, adapted for the different metabolic and growth-stimulating microenvironment within the brain, may undergo stress, when reintroduced to an in vitro culture. Additionally, we cannot exclude an effect of DMSO as it induced cytotoxicity at certain concentrations in vivo [44]. However, much lower DMSO concentration/volume (20 %/200 µl) was injected during this study than was previously reported in ([44] and refs therein).

Previous studies showed that TMZ or radiotherapy with TMZ treatment of glioblastoma cells was associated with a reduced glucose uptake [10, 22]. To test the sensitivity of cell growth to a glucose concentration reduction, proliferation was analyzed in low-glucose medium (1 g/L glucose) and compared to high-glucose medium (4.5 g/L glucose). Proliferation of U251cells did not change, was slightly increased for U251TMZ1 cells but reduced for U251TMZ2 cells. Furthermore, in contrast to high-glucose medium, no significant difference in proliferation was observed between U251, U251TMZ1 and U251TMZ2 cells in low-glucose medium (Fig. 4b). Both T98G and T98GTMZ cells were highly sensitive to a reduction in glucose concentration, demonstrating significantly inhibited, comparable growth. Proliferation of C6 cells was also reduced in low-glucose medium, whereas no change in proliferation was detected for C6TMZ, C6R1 and C6R2TMZ cells (Fig. 4b). Significantly, C6TMZ and C6R1 cells proliferated faster than C6 cells in low-glucose medium, whereas proliferation of C6 and C6R2TMZ cells was comparable (Fig. 4b).

An analysis of colony formation efficiency showed no significant difference between U251 and U251TMZ1 or U251TMZ2 cells; however, U251TMZ2 cells formed more colonies than U251TMZ1 cells. T98GTMZ cells formed more colonies than T98G cells, whereas C6TMZ and C6R2TMZ cells formed a fewer number of colonies than C6 and C6R1 cells, respectively (Fig. 4c). This is in agreement with the previous studies where it was demonstrated that colony formation efficiency or growth in vivo of long-term TMZ-treated cells was increased, decreased or unchanged (Table 1).

**Fig. 5:**
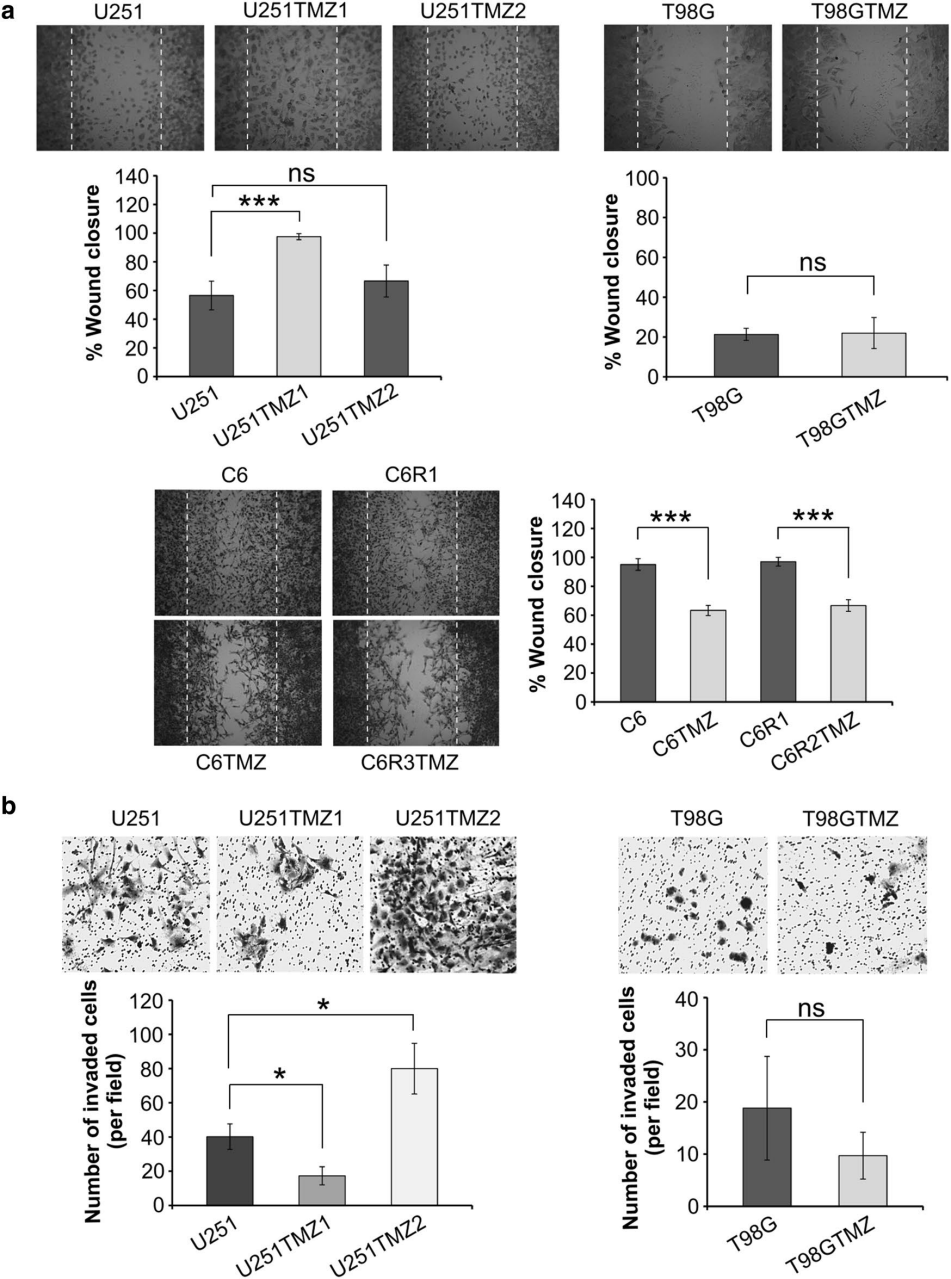
Long-term TMZ treatment promotes diverse changes in migration and invasion. **a** Representative photographs and quantitation of wound closure in scratch wound healing assay. **b** Representative photographs and quantitation of a number of invaded cells in a transwell invasion assay. *P < 0.05; **P < 0.01; ***P < 0.001; *NS* non significant

U251TMZ1 cells migrated faster than U251 cells but no difference in migration was observed between U251 and U251TMZ2 cells (Fig. 5a). Furthermore, no difference in migration was detected between T98G and T98GTMZ cells, whereas C6TMZ and C6R2TMZ cells migrated slower than C6 and C6R1 cells, respectively. In contrast to a migration analysis, transwell invasion assay demonstrated a lower and higher invasion rate of U251TMZ1 and U251TMZ2 cells, respectively (Fig. 5b). Similar to a migration analysis, no difference in invasion rate was observed between T98G and T98GTMZ cells. Similar results were obtained in the previous studies where migration or invasion of long-term TMZ-treated cells was found to be increased, decreased or unaffected (Table 1).

Quantitative real time PCR (qRT-PCR) analysis of the expression of stem cell markers *CD133*, *OCT4*, *SOX2* and *NANOG* showed more than a twofold up-regulation of only *CD133* in U251TMZ1 and U251TMZ2 cells, whereas more than a twofold down-regulation of *CD133* was observed in T98GTMZ cells, *OCT4* in T98GTMZ cells, and *SOX2* in U251TMZ1 cells (Additional file 9: Figure S1a).

**Fig. 6:**
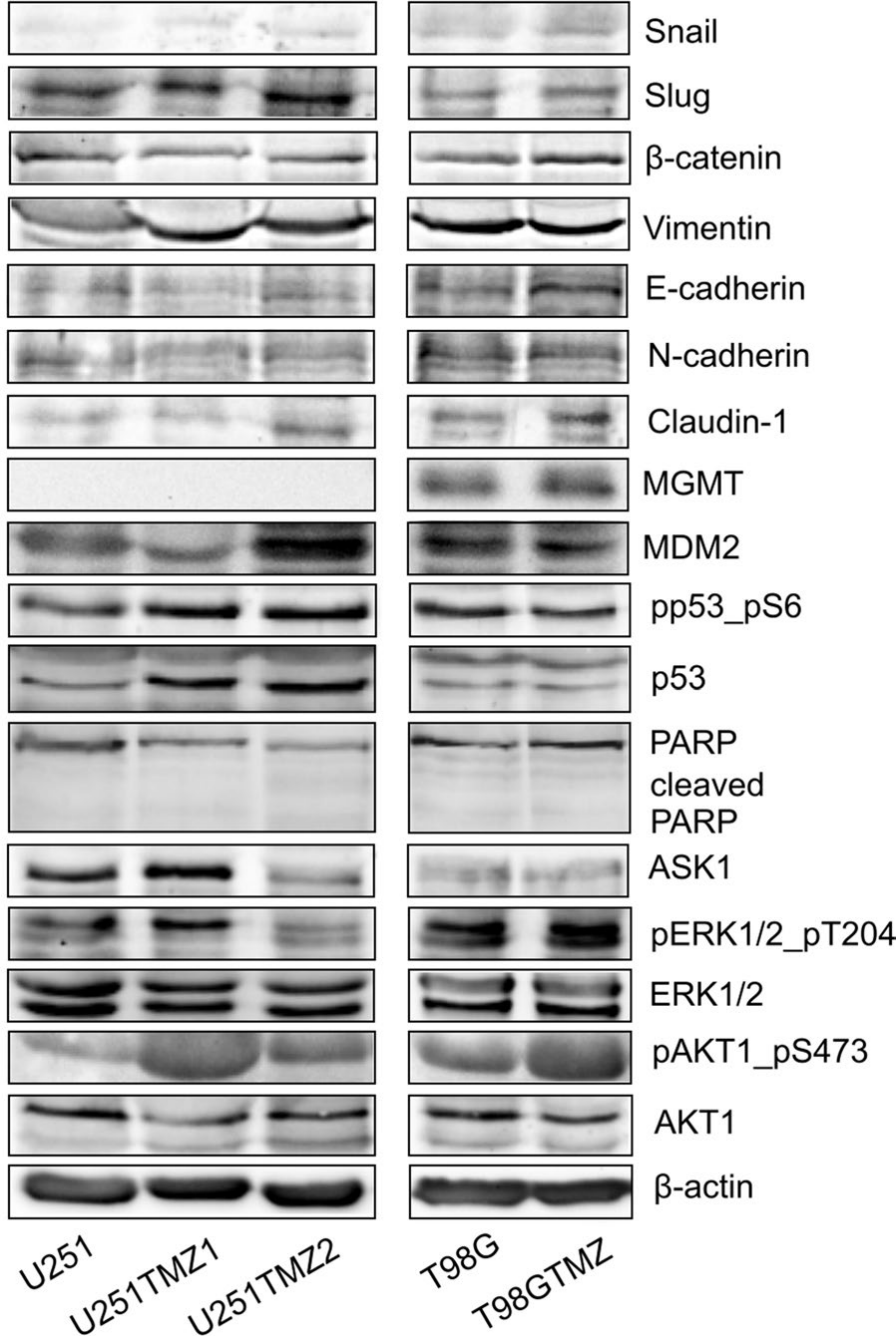
Long-term TMZ-treated cells have an individual pattern of expression/activation of the EMT markers and signal transduction pathway components. Proteins were evaluated by Western blot analysis with specific antibodies. *AKT* v-akt murine thymoma viral oncogene homolog 1, *ASK1* mitogen-activated protein kinase kinase kinase 5, *ERK* mitogen-activated protein kinase, *MGMT* O-6-methylguanine-DNA methyltransferase, *MDM2* MDM2 proto-oncogene, E3 ubiquitin protein ligase, *PARP* poly (ADP-ribose) polymerase 1, *Snail* snail family zinc finger 1, *Slug* snail family zinc finger 2

The TMZ-treated cell lines had individual patterns in expression/activation of signal transduction proteins (Fig. 6). An analysis of epithelial-mesenchymal transition (EMT) markers showed increased expression of Vimentin, Slug and Claudin-1 in U251TMZ2 cells and Vimentin in U251TMZ1 cells. No significant changes in EMT markers expression were revealed between T98G and T98GTMZ cells. U251TMZ2 but not U251TMZ1 cells had increased expression of MDM2. In contrast, U251TMZ1 but not U251TMZ2 cells had increased pAKT1, pERK1/2, and ASK1. T98GTMZ cells had increased pAKT1, pERK but not ASK1 and MDM2. Both U251TMZ1 and U251TMZ2 cells but not T98GTMZ cells had increased total and phosphorylated p53 levels. T98GTMZ cells but not U251TMZ1 or U251TMZ2 cells expressed MGMT. In addition, no *MGMT* expression in U251, U251TMZ1 or U251TMZ2 cells was detected by qRT-PCR (Additional file 9: Figure S1b). No PARP expression changes or cleavage was observed. If we extrapolate this low-scale Western blot analysis data on the whole (phospho)proteome, a striking difference and individuality of each TMZ-treated cell line in comparison to control cells would be revealed as it was demonstrated previously [13–15].

**Fig. 7:**
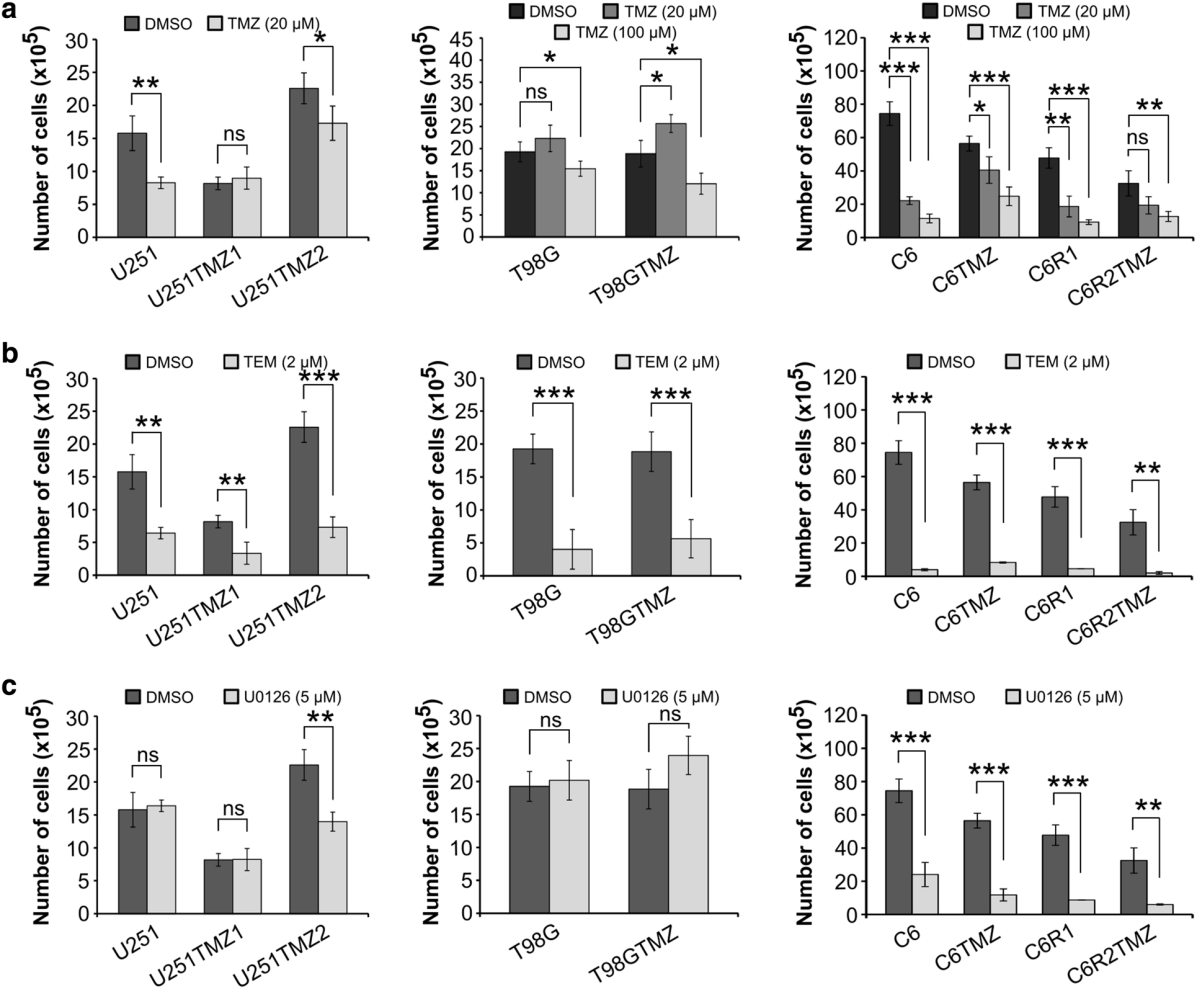
Long-term TMZ treatment increases the resistance to TMZ re-challenge but not to TEM or U0126 treatment. **a**–**c** Proliferation of a vehicle or TMZ (20 or 100 µM), TEM (2 µM) or U0126 (10 µM) treated cells was compared. U251, T98G (5 × 10^4^ cells) and C6 (1 × 10^4^ cells) derivatives were treated for 7 days with a single dose of TMZ, TEM, or U0126 or a vehicle (DMSO did not exceed 0.1 % by volume). Cells viability was evaluated by trypan blue exclusion assay. *P < 0.05; **P < 0.01; ***P < 0.001; *NS* non significant

Finally, we analyzed whether the TMZ-treated cells changed sensitivity to TMZ re-challenge. U251TMZ1 and U251TMZ2 cells were less responsive to 20 µM TMZ. T98GTMZ but not T98G cells grew slightly faster in the presence of 20 µM TMZ, whereas their growth was comparably inhibited by 100 µM TMZ. Proliferation of C6 cells was significantly inhibited by 20 or 100 µM TMZ, whereas the relative ratios of growth inhibition after TMZ re-challenge of C6 derivatives were C6 > C6R1 > C6TMZ ≈ C6R2TMZ (Fig. 7a). All cell lines were highly sensitive to 2 µM temsirolimus (TEM, mTOR kinase inhibitor) with no changes in the sensitivity after long-term TMZ treatment (Fig. 7b). 5 µM U0126 (an extensively studied experimental MEK1/2 inhibitor [45]) inhibited proliferation of U251TMZ2 but not U251 or U251TMZ1 cells. Proliferation of both T98G and T98GTMZ cells was insensitive to U0126. In contrast, C6 derivatives were highly sensitive to U0126 with no change in response after TMZ treatment (Fig. 7c).

## Discussion

In this study we characterized genome-phenotype changes of long-term TMZ-treated glioblastoma cell lines and found that TMZ may either increase or reduce genomic diversity (CCA/NCCAs) and tumour cell aggressiveness. An increase of resistance to TMZ re-challenge seems to be the only fundamental common and predictable trait intrinsic to all long-term TMZ-treated cells; all other phenotype responses were versatile (Table 1). Our data indicate that changes in genome stability and diversity may be responsible for individual and heterogeneous phenotypes of long-term TMZ-treated cells. It is worth emphasizing that U251TMZ1 and U251TMZ2 cell lines, established by parallel selection of the same parental cell line with the same chemotherapy agent under similar treatment conditions with the only difference in the duration of treatment (10 versus 5 weeks), underwent individual genomic and phenotypic evolution. The development of a heterogeneous range of drug-resistant lines with individual genomic and/or phenotypic changes from the same cell line, treated with the same chemotherapy agent (e.g., cisplatin, puromycin) was reported previously [3, 31].

The therapy-driven glioblastoma genome evolution was scarcely reported previously. An analysis of primary cell cultures established from three surgery glioblastoma specimens of the same patient (primary specimen and two consecutive recurrences after lomustine and TMZ therapy) demonstrated a distinct subclonal architecture, abnormalities in karyotypic pattern, and rates of proliferation and migration [46]. Extending research on additional matched primary and recurrent glioblastomas, authors revealed that therapy either increased chromosomal aberrations in some cases that correlated with relatively short overall survival or reduced genome diversity in other cases and these patients showed a much longer overall survival [46]. Recent sequencing of primary and TMZ-treated recurrent gliomas showed the TMZ-driven amplification of mutation heterogeneity (hypermutation phenotype) in IDH1-mutant but not IDH1-wild-type astrocytic gliomas [36–38]. High levels of MGMT methylation and intrinsic or acquired mutations in the key MMR genes and/or MGMT were associated with hypermutation phenotype [36]. However, these studies were primarily focused on alterations in DNA sequence rather than on CIN.

The resistance acquisition to TMZ was widely attributed to O^6^-methylguanine-DNA methyltransferase (MGMT). Despite a relatively low proportion of the TMZ-driven cytotoxic O^6^-methylguanine lesion formation (5 %), the methylated promoter of *MGMT* was considered one of the most robust predictor of TMZ response with inverse correlation [47, 48]. However, this generally good correlation between *MGMT* methylation and TMZ treatment response was recently challenged. The TCGA Research Network reported that *MGMT* promoter methylation could serve as a predictive biomarker only in the glioblastoma classical subtype but not in the other subtypes (mesenchymal, proneural or neural) [49]. Moreover, clonal analysis of glioblastoma samples demonstrated inter-tumor variability in *MGMT* promoter methylation and MGMT protein expression levels, which were inconsistent with TMZ responses [6]. Similarly, no correlation between the TMZ sensitivity and *MGMT* promoter methylation, mRNA or protein expression was revealed for eleven diffuse large B cell lymphoma cell lines [4]. Here we found that long-term TMZ-treated cells reduced sensitivity to TMZ re-challenge without changing MGMT mRNA or protein expression levels. On the other hand, previous reports based on transcriptome analysis elucidated that complex individual genetic networks rather than a specific common mechanism conferred a different TMZ sensitivity [4]. Furthermore, the TMZ-resistant variants of Hs683, U87, and LNZ308 cell lines demonstrated individuality in global miRNA expression, and the integrative miRNA/mRNA network analysis revealed obvious differences in the genetic network in comparison to control cells [12]. A measurement of global kinase activity of five TMZ resistant cell lines revealed no common kinase-driven pathway of TMZ resistance, and two TMZ resistant lines demonstrated extreme kinomic activity differences in comparison to control cells [15]. Altogether, adaptation of tumour cells to long-term TMZ cytotoxicity and genotoxicity is associated with profound diverse changes in the transcriptome, proteome, kinome and metabolome [8–15], the versatile phenotype responses (Table 1), involvement of many proteins/miRNAs (Additional file 8: Table S8) (see also the recent large synthetic lethal screens for “TMZ-sensitizing genes”) [9, 50] and DNA repair pathways [51].

The cancer stem cell hypothesis postulates a significant role of glioblastoma cancer stem cells (GSC) in therapy resistance and tumour recurrence. However, a recent study showed that clones of GSC had distinct tumourigenic potential that was determined by their genetic diversity rather than expression levels of different GSC-associated markers (CD133, CD15, A2B5 or CD44) [52]. Moreover, although TMZ treatment induced conversion of non-GSC into GSC both in vitro and in vivo [53], the majority of patient-matched GSC and non-GSC cultures (25 tested) had a similar TMZ responsiveness and in some cases GSC were even more sensitive [54]. These studies highlight the primary importance of genetic heterogeneity in tumorigenic potential of CSC-associated populations, and furthermore point to dynamic plasticity of tumor cells under TMZ therapy and no superiority of GSC over bulk tumor population in TMZ resistance.

There are approximately twenty current clinical studies using mTOR inhibitors for the treatment of gliomas [55]. However, phase II studies with recurrent glioblastoma reported no efficacy of TEM in the combination with TMZ, sorafenib, bevacizumab, or erlotinib [55]. Although we used a clinically relevant TEM concentration [56], the discrepancy between cell culture responses (Fig. 7b) and patient responses is obvious. It is worth noting that TEM is also able to induce/promote CIN in tumor and normal cells [57]. A targeted therapy failure in patients with recurrent glioblastoma after radiotherapy with TMZ [58] highlights the necessity to lower the evolutionary potential of a tumour and constrain its dynamics by directing efforts at reducing tumour population diversity, at potentiating the immune system and homeostasis of the individual.

In conclusion, our current data improve the knowledge on the TMZ-instigated genome evolution and highlight the primary importance of genetic instability in chemotherapy failure as the more different combinations of molecular mechanisms exist within a cancer cell population, the more likely a population adapts to drug cytotoxicity/genotoxicity. TMZ treatment-associated changes of the genetic network (gene content, RNA and protein expression and their interaction), which are governed by changes of the genome context (number and structure of chromosomes and their nuclear topology) may offer an explanation for why the versatile and opposite phenotype responses of long-term TMZ treated tumor cells were observed in different studies (Table 1). Although our study is limited to the use of established glioblastoma cell lines, our results are consistent with a recent report on evolution of low-grade gliomas to aggressive high-grade glioblastoma in 6 of 10 patient cases due to an increased mutation load upon TMZ therapy [38]. Our results and the latter study suggest that the therapeutic promotion of excessive genetic instability/heterogeneity is a double-edged sword: while the primary response in the form of increased overall survival will be positive, the price for moderate inhibition of tumour growth will be changes in the genomic landscape, tumour subclonal architecture, and, eventually, promotion of cancer evolution, which ultimately impacts the therapeutic management of recurrence.

## Methods

### Cell cultures

Human U251 (Bank of Cell Lines from Human and Animal Tissues, R.E. Kavetsky Institute of Experimental Pathology, Oncology and Radiobiology, Kyiv, Ukraine), T98G (ATCC) and rat C6 (Pirogov Russian State Medical University, Moscow, Russia) glioma cell lines were grown in DMEM (HyClone, Thermo Scientific, UK) supplemented with 10 % fetal bovine serum (FBS, HyClone) and 100 µg/ml penicillin/100 u/ml streptomycin (Sigma, USA) in an environment of 95 % air/5 % CO2. U251, T98G, and C6 cell lines are isocitrate dehydrogenase 1 (IDH1)-wild-type. U251 is MGMT-negative; T98G is MGMT-positive.

### Pharmacological agents

Temozolomide (TMZ, Sigma), Temsirolimus (TEM, Abcam Biochemicals, USA) and U0126 (Abcam Biochemicals) were dissolved in DMSO to a concentration of 100 mM. The final DMSO concentration in the culture medium did not exceed 0.4 %. Stock solutions of all drugs were stored at −20 °C.

### TMZ treatment of glioblastoma cells in vitro

U251, T98G, and C6 glioblastoma cell lines were treated with DMSO or TMZ (Sigma) twice with 25 μM, twice with 50 μM and then with 100 μM TMZ twice per week during 5 weeks (U251TMZ2, T98GTMZ and C6TMZ) or 10 weeks (U251TMZ1), followed by several weeks of washout (in the TMZ-free medium) before in vitro tests. DMSO did not exceed 0.1 % of the culture medium.

### TMZ treatment of C6 cells in vivo

The animals were kept in accordance with the Guidelines on Laboratory Practices adopted by the Ministry of Health of the Russian Federation (Order 267, 19 June 2003). The protocol stipulating animal treatment was approved by the Ethics Committee of N. I. Pirogov Russian State Medical University, and all rules and regulations were followed during experimentation on animals. Glioma modeling was performed by the intracerebral stereotactic implantation (Leica stereotactic device, USA) of C6 cells (5 × 10^5^) into the striatum region of ketamine-anesthetized adult female Wistar rats as described previously [59]. Rats with C6 glioma received 20 % DMSO (n = 1, C6R1) or TMZ (n = 1, C6R2TMZ) injected intraperitoneally (i.p.) three times per week at a dose of 50 mg/kg. Rats were sacrificed after 10 injections. Gliomas were aseptically harvested, mechanically disaggregated, and a cell suspension was seeded into adherent dishes. Cells were used at the passages 3–10 for analysis.

### Conventional cytogenetics

Chromosome samples were prepared as described previously [23]. 200 metaphase plates were calculated for distribution of chromosome across cells. 20 metaphases (U251 and T98G derivatives) or 10 metaphases (C6 derivatives) were described for chromosome abnormalities, according to the International System for Human Cytogenetic Nomenclature (ISCN 2013). Clonal chromosome aberrations (CCAs) were defined as aberrations found at least in two cells among examined metaphases, whereas non-CCAs (NCCAs) as aberrations detected in only one cell. The frequency of NCCAs in a cell line was calculated by dividing the number of metaphases displaying NCCAs to the total number of examined metaphases (×100 %). Only structural NCCAs were considered.

### Array comparative genome hybridization (aCGH)

A total DNA was isolated using NucleoSpin Blood DNA extraction kit (Macherey–Nagel, Germany) according to the manufacturer’s instructions. To analyze copy number alterations (CNAs), aCGH was performed as detailed previously [23]. Human and rat cell lines were analyzed on the CytoSure Aneuploidy Array 15 k (Oxford Gene Technologies, UK) and 180 K microarrays (Agilent Technologies, USA), respectively. Image analysis of human and rat samples was carried out with CytoSure Analysis Software (Oxford Gene Technologies) and Agilent CytoGenomics Edition 2.9.2.4, respectively.

### Cell proliferation in a high and low-glucose medium

Cells were seeded onto 6 cm dishes at a density of 5 × 10^4^ (U251 and T98G derivatives) or 1 × 10^4^ (C6 derivatives) and grown in high-glucose (4.5 g/L) or low-glucose (1 g/L) DMEM with 10 % FBS. On the 7th day of seeding, cells were harvested, incubated with trypan blue, and calculated using hemocytometer. Experiments were repeated at least three times.

### Cell viability test

U251, T98G (5 × 10^4^ cells) and C6 (1 × 10^4^ cells) derivatives were seeded onto 6 cm dishes and incubated overnight. The cells were treated for 7 days with a single dose of TMZ (20 and 100 µM), TEM (2 µM), U0126 (5 µM) or DMSO. Experiments were repeated at least three times. Cell viability was evaluated by trypan blue exclusion assay instead of metabolically-based MTT or ATP assays, which are prone to over/underestimate cell viability under cytotoxic stress [45].

### Soft agar colony formation assay

5 × 10^3^ cells were placed in 1.5 ml of 0.35 % low gelling temperature agarose (Gibco, Life Technologies, USA) with DMEM supplemented with 10 % FBS. 0.35 % top agarose was poured on 1.5 ml of solidified 0.5 % base agarose/10 % FBS/DMEM. Cells were seeded in triplicates in a 35-mm dish and grown at 37 °C for 21 days to allow colony formation. Colonies were visualized by staining with 0.005 % crystal violet, photographed, counted using OpenCFU software [60], and expressed as the means of triplicates of four independent experiments.

### Scratch wound healing assay

Using a P200 pipette tip, the scratches were made by scraping across the confluent cell monolayer. Pictures were taken at 0 and 16 h (C6 derivatives) or 24 h (U251 and T98G derivatives) and automated image analysis was carried out using TScratch software [61] to avoid any potential bias in quantifying an extent of migration. At least twelve wound healing areas for each cell line were photographed and analyzed to take into account the differences in cell density and widths of scratches. The per cent of wound area closure was calculated taking open wound area at 0 h for 100 %.

### Cell invasion assay

A 24-well tissue culture plate-based Chemicon cell invasion assay (QCM ECMatrix 550, Millipore, USA) was performed according to the manufacturer’s protocol. 2 × 10^5^ cells were seeded to the inserts. After 24 h, five fields of invaded cells in each well were randomly photographed and counted manually. Test was performed two times.

### Real time quantitative PCR

Total RNA was extracted from cell lines using TRI Reagent (Sigma, #T9424) according to the manufacturer’s recommendations. Equal amounts of total RNA (5 μg for 20 μl reaction mixture) were transcribed into cDNA with random hexamer primers and RevertAid Reverse Transcriptase (Thermo Scientific, #EP0441). Twofold diluted cDNA and gene specific primers were mixed with Maxima SYBR Green qPCR Master Mix (2X) (Fermentas, #K0251) according to the manufacturer’s recommendations. qRT-PCR was run in triplicates on CFX96 RT-PCR Detection System (Bio-Rad). The amplification procedure of target genes was as follows: initial denaturing step at 95 °C for 10 min, followed by 40 cycles of denaturation at 95 °C for 15 s, annealing at 59 °C for 30 s and extension at 72 °C for 30 s. Melting curve analysis was performed to confirm amplification specificity. To calculate the relative gene expression ratios (fold-change), C_T_ method (also known as the 2^−ΔΔCT^ method, expressed as ratios relative to control values after normalization to the internal control *TBP*—TATA-binding protein) was applied. C_T_ values were derived using Bio-Rad CFX Manager 3.1.

### Primers

*TBP* Forward: TGCACAGGAGCCAAGAGTGAA; Reverse: CACATCACAGCTCCCCACCA; *CD133* Forward: CGTGGATGCAGAACTTGACAACGT; Reverse: ATACCTGCTACGACAGTCGTGGT; *SOX2* Forward: GCCGAGTGGAAACTTTTGTCGGA; Reverse: CGTGTACTTATCCTTCTTCATGAGCGTC; *OCT4* Forward: GGAGAAGGAGAAGCTGGAGCA; Reverse: GGCAGATGGTCGTTTGGCTGAATA; *NANOG* Forward: GTCTGGACACTGGCTGAATCCT; Reverse: CTCGCTGATTAGGCTCCAACCAT; *MGMT* Forward: CCTGGCTGAATGCCTATTTCCACCA; Reverse: GGATGAGGATGGGGACAGGATTGC.

### Western blot analysis

Total cell lysates were analyzed as described earlier [23]. The following antibodies were used: mouse anti-MGMT (Novus Biologicals; #NB100-168), rabbit anti-PTCH2 (Cell Signaling; #2464), rabbit anti-ASK1 (Cell Signaling; #8662), rabbit anti-MDM2 (Thermo Fisher Scientific Pierce; #PA5-11353), rabbit anti-p53 (Millipore; #04-1083), rabbit anti-phospho-p53 (Ser6) (Millipore; #04-540), rabbit anti-PARP (Cell Signaling; #9542), rabbit anti-ERK1/2 (Millipore; #06-182), rabbit anti-phospho-ERK1/2 (Thermo Scientific Pierce; #MA5-1574), rabbit anti-AKT1 (Millipore; #07-416), mouse anti-phospho-AKT1 (Santa Cruz; sc-52940), mouse anti-β-actin (Sigma-Aldrich; A1978), the Epithelial-Mesenchymal Transition antibody sampler kit (Cell Signaling; #9782), anti- rabbit (Cell Signaling, #7074) and anti-mouse (Cell Signaling, #44209).

### Statistical analyses

A two-sided t test was used to calculate the significance values (Statistica 10 Software, USA). Data showing p values of *P < 0.05, **P < 0.01, and ***P < 0.001 were considered significant. All experimental data are reported as mean and the error bars represent the experimental standard error (±standard deviation, SD).

## Additional files

10.1186/s12935-016-0311-8A list of CCAs/NCCAs and their copy number variation in U251, U251TMZ1 and U251TMZ1 cells.
10.1186/s12935-016-0311-8Table S2. Detailed description of copy number alterations (CNAs) in U251, U251TMZ1 and U251TMZ1 cells.
10.1186/s12935-016-0311-8Table S3. A list of CCAs/NCCAs and their copy number variation in T98G and T98GTMZ cells.
10.1186/s12935-016-0311-8Table S4. Detailed description of copy number alterations (CNAs) in T98G and T98GTMZ cells.
10.1186/s12935-016-0311-8Table S5. A list of CCAs/NCCAs and their copy number variation in C6, C6TMZ, C6R1, and C6R2TMZ cells.
10.1186/s12935-016-0311-8Table S6. Detailed description of copy number alterations (CNAs) in C6 and C6TMZ cells.
10.1186/s12935-016-0311-8Table S7. A list of annotated genes in gained 4p16.1-q26 region retrieved using a the DAVID bioinformatics resource or b the NCBI Map Viewer. These lists of annotated genes were cross-checked with a manually curated list of published proteins/miRNAs that were shown to contribute to TMZ resistance (see Suppl. Table S8), and hits were marked with colour.
10.1186/s12935-016-0311-8Table S8. A list of genes/proteins, which were experimentally shown to increase/reduce the sensitivity of tumour cells to temozolomide (TMZ) treatment.
10.1186/s12935-016-0311-8qRT-PCR analysis of selected genes in TMZ-treated cells. a The relative gene expression ratios (fold-change) of stem cell markers CD133, OCT4, SOX2, and NANOG. b The relative gene expression ratio (fold-change) of MGMT. Gene expression fold-change values were derived by 2^-ΔΔCT^ method (expressed as ratios relative to control values after normalization to the internal control TBP). TBP – TATA-binding protein; CD133 – prominin 1; OCT4 – POU class 5 homeobox 1; SOX2 – SRY (sex determining region Y)-box 2; NANOG – Nanog homeobox; MGMT – O-6-methylguanine-DNA methyltransferase.

## Abbreviations

aCGH: array comparative genome hybridization
CIN: chromosome instability
CCAs: clonal chromosome aberrations
CNAs: copy number alterations
GSC: glioblastoma cancer stem cells
NCCAs: non-clonal chromosome aberrations
TEM: temsirolimus
TMZ: temozolomide
