# Supplementary material for: Temozolomide promotes genomic and phenotypic changes in glioblastoma cells
Source: Cancer Cell Int. 2016 May 5;16:36. doi: 10.1186/s12935-016-0311-8 (PMC4858898; doi:10.1186/s12935-016-0311-8)
Supplement: Supplementary file 8 — 10.1186/s12935-016-0311-8Table S8. A list of genes/proteins, which were experimentally shown to increase/reduce the sensitivity of tumour cells to temozolomide (TMZ) treatment. [file 12935_2016_311_MOESM8_ESM.doc]

| **Supplementary Table 8. Genes/proteins, which were experimentally shown to increase/reduce the sensitivity of tumour cells to temozolomide (TMZ) treatment** | | | |
| --- | --- | --- | --- |
| **Gene** | **Protein** | **Suggested manipulation to increase TMZ efficiency** | **Reference** |
| *ADAM8* | A disintegrin and metallopeptidase domain 8 | ↓ | Dong et al., 2015 |
| *AGT* | O6-alkylguanine-DNA-alkyltransferase | ↓ | Fontijn et al., 2007; Ma et al., 2002 |
| *AJAP1* | Adherens junction-associated protein 1 | ↑ | Zeng et al., 2014 |
| *AKR1C* | Aldo-keto reductase family 1 | ↓ | Le Calvé et al., 2010 |
| *AKT* | V-akt murine thymoma viral oncogene homolog | ↓ | Chen et al., 2012; Hirose et al., 2005; Turner et al., 2015 |
| *ALDH1A1* | Aldehyde dehydrogenase 1A1 | ↓ | Schäfer et al., 2012 |
| *ALKBH2* | The DNA repair protein AlkB homolog 2 | ↓ | Johannessen et al., 2012 |
| *ANXA5* | Annexin A5 | ↓ | Wu et al., 2014 |
| *APEX1* | Apurinic/apyrimidinic endonuclease 1 | ↓ | McNeill et al., 2009; Silber et al., 2002 |
| *APNG* | Alkylpurine-DNA-N-glycosylase | ↓ | Agnihotri et al., 2012 |
| *AURKA* | Aurora kinase | ↓ | Xie and Meyskens, 2013 |
| *AXL* | AXL receptor tyrosine kinase | ↓ | Keating et al., 2010 |
| *Bak1* | Bcl-2 antagonist killer 1 | ↑ | Chen et al., 2014 |
| *BCL2* | B-cell CLL/lymphoma 2 | ↓ | Voss et al., 2010 |
| *BIRC* | Baculoviral IAP repeat containing 5 | ↓ | Virrey et al., 2008 |
| *BMP2* | Bone morphogenetic protein 2 | ↑ | Persano et al., 2012 |
| *BNIP3* | BCL2/adenovirus E1B 19kDa interacting protein 3 | ↓ | Burton et al., 2009 |
| *BRCA2* | Breast cancer 2 | ↓ | Kondo et al., 2011; Quiros et al., 2011 |
| *BUB1; BUBR1* | BUB1 mitotic checkpoint serine/threonine kinase; Bub1-related kinase | ↓ | Morales et al., 2013 |
| *CAV1* | Caveolin-1 | ↑ | Quann et al., 2013 |
| *CCN2* | Connective tissue growth factor | ↓ | Yin et al., 2010 |
| *CD74* | Major histocompatibility complex, class II invariant chain | ↓ | Kitange et al., 2010 |
| *CDC2* | Cyclin-dependent kinase | ↓ | Hayashi et al., 2013 |
| *CDK6* | Cyclin-dependent kinase 6 | ↓ | Li et al., 2012 |
| *CHI3L1* | Chitinase 3-like 1 (cartilage glycoprotein-39) | ↓ | Akiyama et al., 2014 |
| *CHK1* | Checkpoint kinase 1 | ↓ | Hirose et al., 2001 |
| *CNO* | Cappuccino protein | ↓ | Huang et al., 2012 |
| *COOL1* | Rho guanine nucleotide exchange factor (GEF) 7 | ↓ | Stevens et al., 2014 |
| *COX* | Cytochrome c oxidase | ↓ | Oliva et al., 2010 |
| *CX43* | Connexin 43, gap junction protein | ↓ | Gielen et al., 2013 |
| *CXCL12/SDF1* | Chemokine stromal cell-derived factor-1 | ↓ | Hattermann et al., 2012 |
| *DCR1* | Decoy Receptor 1 | ↓ | Mansour et al., 2015 |
| *DNMT* | DNA methyltransferase | ↓ | [Shervington A](http://www.ncbi.nlm.nih.gov/pubmed?term=Shervington A%5BAuthor%5D&cauthor=true&cauthor_uid=18928331), [Patel R](http://www.ncbi.nlm.nih.gov/pubmed?term=Patel R%5BAuthor%5D&cauthor=true&cauthor_uid=18928331)., 2008 |
| *DR5* | Death receptor 5 | ↓ | Fiveash et al., 2008 |
| *EFEMP1* | EGF containing fibulin-like extracellular matrix protein 1 | ↓ | Hiddingh et al., 2013 |
| *FANCD2* | Fanconi anemia, complementation group D2 | ↓ | Chen et al., 2007 |
| *FoxM1* | Forkhead box M1 | ↓ | Zhang et al., 2012 |
| *FRP4* | Frizzled-related protein 4 | ↑ | G et al., 2015 |
| *GAL1* | Galectin 1 | ↓ | Le Mercier et al., 2008; Mathieu et al., 2007 |
| *GART* | Glycinamide ribonucleotide formyl transferase | ↓ | Liu et al., 2013 |
| *GATA4* | GATA binding protein 4 | ↑ | [Agnihotri](http://www.ncbi.nlm.nih.gov/pubmed?term=Agnihotri S%5BAuthor%5D&cauthor=true&cauthor_uid=21464220) et al., 2011 |
| *GLS2* | Liver-type glutaminase | ↑ | Szeliga et al., 2012 |
| *GLUT3* | Glucose transporter type 3 | ↓ | Le Calvé et al., 2010 |
| *GRM3* | Glutamate receptor metabotropic | ↓ | Ciceroni et al., 2013 |
| *GRP78* | Heat shock 70kDa protein 5 | ↓ | Virrey et al., 2008; Pyrko et al., 2007 |
| *GSK3B* | Glycogen synthase kinase 3β | ↓ | Pyko et al., 2013 |
| *HIF1A* | Hypoxia-inducible factor-1α | ↓ | Persano et al., 2012; Chen W et al., 2013 |
| *HK2* | Hexokinase 2 | ↓ | Wolf et al., 2011 |
| *HMGB2* | High mobility group box 2 | ↓ | Wu et al., 2013 |
| *HOXA10* | Homeobox A10 | ↓ | Kim et al., 2015 |
| *HOXA9* | Homeobox A9 | ↓ | Pojo et al., 2015 |
| *HSP27; HSP72* | Heat shork protein 27 kDa and 72 kDa | ↓ | Jakubowicz-Gil et al., 2013; Sang et al., 2014 |
| *IAP* | Inhibitor of apoptosis protein | ↓ | Ziegler et al., 2011 |
| *IDH1* | Isocytrate dehydrogenase 1 | ↓ | Wang et al., 2014 |
| *IDO1* | Indoleamine 2,3-dioxygenase | ↓ | Miyazaki et al., 2009 |
| *IFNB* | Interferon beta | ↑ | Roos et al., 2011; Rozati et al., 2008; Happold et al., 2014 |
| *IGF1R* | Insulin-like growth factor 1 receptor | ↓ | Geoerger et al., 2010 |
| *IGF2BP1* | Insulin-like growth factor 2 mRNA binding protein 1 | ↓ | [Craig EA](http://www.ncbi.nlm.nih.gov/pubmed?term=Craig EA%5BAuthor%5D&cauthor=true&cauthor_uid=21981993), [Spiegelman VS](http://www.ncbi.nlm.nih.gov/pubmed?term=Spiegelman VS%5BAuthor%5D&cauthor=true&cauthor_uid=21981993) 2012 |
| *IL24* | Interleukine 24 | ↑ | Zheng et al., 2008 |
| *IL8* | Interleukine 8 | ↓ | Luo et al., 2012 |
| *IRAK4* | Interleukin-1 receptor associated kinase- 4 |  | Kumar et al., 2013 |
| *ITGA5* | Integrin alpha 5 | ↓ | Janouskova et al., 2012; Niibori-Nambu et al., 2013 |
| *ITGB4* | Integrin beta 4 | ↓ | Li et al., 2013 |
| *JNK* | c-Jun NH2-terminal kinase | ↓ | Okada et al., 2014 |
| *KEAP1* | Kelch-like ECH-associated protein 1 | ↓ | Zhou et al., 2013 |
| *KLK6* | Kallikrein 6 | ↓ | Drucker et al., 2013 |
| *L1CAM* | L1 cell adhesion molecule | ↓ | Held-Feindt et al., 2012 |
| *LIG4* | DNA ligase IV | ↓ | Kondo et al., 2009 |
| *LRIG1* | Leucine-rich repeats and immunoglobulin-like domains 1 | ↑ | Qi et al., 2013 |
| *MAPK14* | Mitogen-activated protein kinase 14, p38α | ↓ | Hirose et al., 2003 |
| *MCL1* | Myeloid cell leukemia 1 | ↓ | Gratas et al., 2014 |
| *MDM2* | MDM2 proto-oncogene, E3 ubiquitin protein ligase | ↓ | Costa et al., 2013 |
| *MEK* | Mitogen-activated protein kinase kinase | ↓ | Sato et al., 2011 |
| *MGMT* | O-6-methylguanine-DNA methyltransferase | ↓ | Passagne et al., 2006 |
| *miR-124* |  | ↑ | Shi et al., 2014 |
| *miR-125b* |  | ↓ | Wan et al., 2013 |
| *miR-125b* |  | ↓ | Chen et al., 2014; Haemmig et al., 2014 |
| *miR-125b-2* |  | ↓ | Shi et al., 2012 |
| *miR-130a* |  | ↑ | Chen et al., 2015 |
| *miR-136* |  | ↑ | Wu et al., 2015 |
| *miR-139* |  | ↑ | Li et al., 2013 |
| *miR-17* |  | ↓ | Comincini et al., 2013 |
| *miR-181a/b/c/d* |  | ↑ | She et al., 2014; Wang et al., 2013 |
| *miR-181d, miR-767-3p, miR-648* |  | ↑ | Kreth et al., 2013 |
| *miR-195* |  | ↓ | Ujifuku et al., 2010 |
| *miR-21* |  | ↓ | Wong et al., 2012; Zhang et al., 2012 |
| *miR-210* |  | ↑ | Lee et al., 2015 |
| *miR-211* |  | ↑ | Asuthkar et al., 2012 |
| *miR-381* |  | ↓ | Wang et al., 2015 |
| *miR-9* |  | ↓ | Munoz et al., 2013 |
| *MKP1* | MAP Kinase Phosphatase-1 | ↓ | Yu et al., 2012 |
| *MLH1* | MutL homolog 1 | ↑ | Shinsato et al., 2013 |
| *MMP14* | Matrix metallopeptidase 14 | ↓ | Ulasov et al., 2013 |
| *MPG* | N-methylpurine DNA glycosylase | ↑ | Tang et al., 2011; Trivedi et al., 2008; |
| *MRP1* | Multiple drug resistance-associated protein 1 | ↓ | Peigñan et al., 2011 |
| *MSH6* | MutS homolog 6 | ↑ | Yip et al., 2009 |
| *MTN* | Melatonin | ↑ | Martín et al., 2013 |
| *NBN* | Nijmegen breakage syndrome protein | ↓ | Eich et al., 2010 |
| *NDRG1* | N-myc downstream regulated gene 1 | ↓ | Weiler et al., 2014 |
| *NEFL* | Neurofilament, light polypeptide | ↑ | Wang et al., 2015 |
| *NFKB1* | Nuclear factor of kappa light polypeptide gene enhancer in B-cells 1 | ↑↓ | Huang et al., 2012; Brassesco et al., 2013 |
| *NMP* | Nucleophosmin | ↓ | Gimenez et al., 2012 |
| *NRF2* | The NF-E2-related factor 2 | ↓ | Zhou et al., 2012; Cong et al., 2014 |
| *NTN4* | Netrin 4 | ↓ | Li et al., 2013 |
| *P4HB* | Prolyl 4-hydroxylase, beta polypeptide | ↓ | Sun et al., 2013 |
| *PARG* | Poly (ADP-ribose) glycohydrolase | ↓ | Tang et al., 2011 |
| *PARP* | Poly-(ADP-ribose) polymerase | ↓ | Tang et al., 2011; [Javle M](http://www.ncbi.nlm.nih.gov/pubmed?term=Javle M%5BAuthor%5D&cauthor=true&cauthor_uid=22084640), [Curtin NJ](http://www.ncbi.nlm.nih.gov/pubmed?term=Curtin NJ%5BAuthor%5D&cauthor=true&cauthor_uid=22084640)., 2011; Horton et al., 2009; Tentori et al., 2008 |
| *PGF* | [Placenta growth factor](http://www.ncbi.nlm.nih.gov/pubmed/21109946) | ↓ | Levati et al., 2011 |
| *PGRN* | Progranulin | ↓ | Bandey et al., 2014 |
| *PHF6* | Plant homeodomain (PHD)-like finger 6 | ↓ | Hiddingh et al., 2014 |
| *PIK3CA* | Phosphatidylinositol-4,5-bisphosphate 3-kinase, catalytic subunit alpha | ↓ | Chen et al., 2012 |
| *PLK1* | Polo-like kinase 1 | ↓ | Pezuk et al., 2013 |
| *PMS2* | PMS2 postmeiotic segregation increased 2 | ↑ | Shinsato et al., 2013 |
| *PODXL* | Podocalyxin | ↓ | Wu et al., 2013; Huang et al., 2013 |
| *POLB* | DNA polymerase β | ↓ | Tang et al., 2011; Trivedi et al., 2008 |
| *PP2A* | Serine/threonine protein phosphatase 2A | ↓ | Martiniova et al., 2011 |
| *PRDX1* | Peroxiredoxin 1 | ↓ | Dittmann et al., 2012 |
| *PRMT5* | Protein arginine methyltransferase | ↓ | Yan et al., 2014 |
| *PRNP* | Prion protein | ↓ | Zhuang et al., 2012 |
| *PTCH1* | Patched 1 | ↓ | Bidet et al., 2012 |
| *PTPRK* | Protein tyrosine phosphatase receptor type kappa gene | ↑ | Agarwal et al., 2013 |
| *RAD51* | RAD51 recombinase | ↓ | Quiros et al., 2011 |
| *REV3L* | REV3-like polymerase | ↓ | Roos et al., 2009 |
| *RLIP76* | Leucine-rich repeats and immunoglobulin-like domains 1 | ↓ | Wang et al., 2013 |
| *ROCK2* | Rho-associated, coiled-coil containing protein kinase 2 | ↓ | Wen et al., 2014 |
| *RR* | Ribonucleotide reductase | ↓ | Figul et al., 2003 |
| *RRAD* | GTP-binding protein RAS associated with diabetes | ↓ | Yeom et al., 2015 |
| *S1P* | Sphingosine-1-phosphate | ↓ | Riccitelli et al., 2013 |
| *STAT3* | Signal transducer and activator of transcription 3 | ↓ | Kohsaka et al., 2012 |
| *TAZ* | Transcriptional co-activator with PDZ-binding motif | ↓ | Tian et al., 2015 |
| *TOP2A* | Topoisomerase (DNA) II alpha 170kDa | ↑ | Arivazhagan et al., 2012 |
| *TP53* | Tumor protein p53 | ↓↑ | Wang et al., 2012; Zheng et al., 2008; Krzyzankova et al., 2012; Srivenugopal et al., 2001 |
| *TRAIL* | Tumor necrosis factor-related apoptosis-inducing ligand | ↓ | Fiveash et al., 2008 |
| *TRF2* | Telomere repeat-binding factor 2 | ↓ | Bai et al., 2014 |
| *TRIM24* | Tripartite motif-containing 24 | ↓ | Zhang et al., 2014 |
| *TRKB* | Tropomyosin receptor kinase | ↓ | Croucher et al., 2015 |
| *TRP2* | Tyrosinase-related protein 2 | ↓ | Liu et al., 2005 |
| *TSPN8* | Tetraspin 8 | ↓ | Pan et al., 2015 |
| *UGCG* | UDP-glucose ceramide glucosyltransferase | ↓ | Giussani et al., 2012 |
| *ULK2* | Unc-51 like autophagy activating kinase 2 | ↑ | Shukla et al., 2014 |
| *UNG, MYH, UBE3B, ICMT; MPG* | Uracil DNA glycosylase; A/G-specific adenine DNA glycosylase; ubiquitin protein ligase E3B; Isoprenylcysteine carboxyl methyltransferase; N-methylpurine DNA glycosylase | ↓ | Svilar et al., 2012 |
| *VAMP8* | Vesicle-associated membrane protein 8 | ↓ | Chen et al., 2015 |
| *VEGFR2* | Vascular endothelial growth factor receptor 2 | ↓ | Kessler et al., 2015 |
| *VPS33A* | Vacuolar protein sorting A protein | ↓ | Huang et al., 2012 |
| *WRN* | Werner syndrome, RecQ helicase-like | ↓ | Blank et al., 2004 |
| *XRCC2* | X-ray repair complementing defective repair in Chinese hamster cells 2 | ↓ | Tsaryk et al., 2006 |
| *YBX1* | Y-box binding protein-1 | ↓ | Gao et al., 2009 |
| *ZEB1* | Zinc finger E-box binding homeobox 1 | ↓ | Siebzehnrubl et al., 2013 |

**References to Supplementary Table 8**

# Agarwal S, Al-Keilani MS, Alqudah MA, Sibenaller ZA, Ryken TC, Assem M. Tumor derived mutations of protein tyrosine phosphatase receptor type k affect its function and alter sensitivity to chemotherapeutics in glioma. PLoS One. 2013 May 16;8(5):e62852. doi: 10.1371/journal.pone.0062852. Print 2013.

# Agnihotri S, Gajadhar AS, Ternamian C, Gorlia T, Diefes KL, Mischel PS, Kelly J, McGown G, Thorncroft M, Carlson BL, Sarkaria JN, Margison GP, Aldape K, Hawkins C, Hegi M, Guha A. Alkylpurine-DNA-N-glycosylase confers resistance to temozolomide in xenograft models of glioblastoma multiforme and is associated with poor survival in patients. J Clin Invest. 2012 Jan 3;122(1):253-66. doi: 10.1172/JCI59334. Epub 2011 Dec 12.

# Agnihotri S1, Wolf A, Munoz DM, Smith CJ, Gajadhar A, Restrepo A, Clarke ID, Fuller GN, Kesari S, Dirks PB, McGlade CJ, Stanford WL, Aldape K, Mischel PS, Hawkins C, Guha A. A GATA4-regulated tumor suppressor network represses formation of malignant human astrocytomas. J Exp Med. 2011 Apr 11;208(4):689-702. doi: 10.1084/jem.20102099. Epub 2011 Apr 4.

# Akiyama Y1, Ashizawa T1, Komiyama M1, Miyata H1, Oshita C1, Omiya M1, Iizuka A1, Kume A1, Sugino T2, Hayashi N3, Mitsuya K3, Nakasu Y3, Yamaguchi K1. YKL-40 downregulation is a key factor to overcome temozolomide resistance in a glioblastoma cell line. Oncol Rep. 2014 Jul;32(1):159-66. doi: 10.3892/or.2014.3195. Epub 2014 May 16.

# Arivazhagan A1, Kumar DM, Sagar V, Patric IR, Sridevi S, Thota B, Srividya MR, Prasanna K, Thennarasu K, Mondal N, Hegde AS, Chandramouli BA, Santosh V, Rao MR, Kondaiah P, Somasundaram K. Higher topoisomerase 2 alpha gene transcript levels predict better prognosis in GBM patients receiving temozolomide chemotherapy: identification of temozolomide as a TOP2A inhibitor. J Neurooncol. 2012 Apr;107(2):289-97. doi: 10.1007/s11060-011-0758-3. Epub 2011 Nov 19.

# Asuthkar S, Velpula KK, Chetty C, Gorantla B, Rao JS. Epigenetic Regulation of miRNA-211 by MMP-9 Governs Glioma Cell Apoptosis, Chemosensitivity and Radiosensitivity. Oncotarget. 2012 Nov;3(11):1439-54.

# Bai Y1, Lathia JD, Zhang P, Flavahan W, Rich JN, Mattson MP. Molecular targeting of TRF2 suppresses the growth and tumorigenesis of glioblastoma stem cells. Glia. 2014 Jun 7. doi: 10.1002/glia.22708. [Epub ahead of print]

# Bandey I1, Chiou SH2, Huang AP3, Tsai JC4, Tu PH1. Progranulin promotes Temozolomide resistance of glioblastoma by orchestrating DNA repair and tumor stemness. Oncogene. 2014 May 5. doi: 10.1038/onc.2014.92. [Epub ahead of print]

# Bidet M, Tomico A, Martin P, Guizouarn H, Mollat P, Mus-Veteau I. The Hedgehog receptor patched functions in multidrug transport and chemotherapy resistance. Mol Cancer Res. 2012 Nov;10(11):1496-508. doi: 10.1158/1541-7786.MCR-11-0578. Epub 2012 Jul 2.

# Blank A1, Bobola MS, Gold B, Varadarajan S, D Kolstoe D, Meade EH, Rabinovitch PS, Loeb LA, Silber JR. The Werner syndrome protein confers resistance to the DNA lesions N3-methyladenine and O6-methylguanine: implications for WRN function. DNA Repair (Amst). 2004 Jun 3;3(6):629-38.

# Brassesco MS, Roberto GM, Morales AG, Oliveira JC, Delsin LE, Pezuk JA, Valera ET, Carlotti CG Jr, Rego EM, de Oliveira HF, Scrideli CA, Umezawa K, Tone LG. Inhibition of NF- κ B by Dehydroxymethylepoxyquinomicin Suppresses Invasion and Synergistically Potentiates Temozolomide and γ -Radiation Cytotoxicity in Glioblastoma Cells. Chemother Res Pract. 2013;2013:593020. doi: 10.1155/2013/593020. Epub 2013 Feb 21.

# Burton TR, Eisenstat DD, Gibson SB. BNIP3 (Bcl-2 19 kDa interacting protein) acts as transcriptional repressor of apoptosis-inducing factor expression preventing cell death in human malignant gliomas. J Neurosci. 2009 Apr 1;29(13):4189-99. doi: 10.1523/JNEUROSCI.5747-08.2009.

# Chen CC, Taniguchi T, D'Andrea A. The Fanconi anemia (FA) pathway confers glioma resistance to DNA alkylating agents. J Mol Med (Berl). 2007 May;85(5):497-509. Epub 2007 Jan 13.

# Chen H1, Li X2, Li W3, Zheng H4. miR-130a can predict response to temozolomide in patients with glioblastoma multiforme, independently of O6-methylguanine-DNA methyltransferase. J Transl Med. 2015 Feb 21;13:69. doi: 10.1186/s12967-015-0435-y.

# Chen J1, Fu X, Wan Y, Wang Z, Jiang D, Shi L. miR-125b inhibitor enhance the chemosensitivity of glioblastoma stem cells to temozolomide by targeting Bak1. Tumour Biol. 2014 Mar 19. [Epub ahead of print]

# Chen L, Han L, Shi Z, Zhang K, Liu Y, Zheng Y, Jiang T, Pu P, Jiang C, Kang C. LY294002 enhances cytotoxicity of temozolomide in glioma by down-regulation of the PI3K/Akt pathway. Mol Med Report. 2012 Feb;5(2):575-9. doi: 10.3892/mmr.2011.674. Epub 2011 Nov 11.

# Chen W, Xiao Z, Zhao Y, Huang L, Du G. HIF-1α inhibition sensitizes pituitary adenoma cells to temozolomide by regulating MGMT expression. Oncol Rep. 2013 Aug 22. doi: 10.3892/or.2013.2689. [Epub ahead of print]

# Chen Y1, Meng D1, Wang H1, Sun R1, Wang D1, Wang S1, Fan J1, Zhao Y1, Wang J1, Yang S1, Huai C1, Song X1, Qin R1, Xu T1, Yun D1, Hu L1, Yang J1, Zhang X1, Chen H1, Chen J1, Chen H1, Lu D1. VAMP8 facilitates cellular proliferation and temozolomide resistance in human glioma cells. Neuro Oncol. 2014 Sep 10. pii: nou219. [Epub ahead of print]

# Ciceroni C, Bonelli M, Mastrantoni E, Niccolini C, Laurenza M, Larocca LM, Pallini R, Traficante A, Spinsanti P, Ricci-Vitiani L, Arcella A, De Maria R, Nicoletti F, Battaglia G, Melchiorri D. Type-3 metabotropic glutamate receptors regulate chemoresistance in glioma stem cells, and their levels are inversely related to survival in patients with malignant gliomas. Cell Death Differ. 2012 Nov 23. doi: 10.1038/cdd.2012.150. [Epub ahead of print]

# Comincini S, Allavena G, Palumbo S, Morini M, Durando F, Angeletti F, Pirtoli L, Miracco C. microRNA-17 regulates the expression of ATG7 and modulates the autophagy process, improving the sensitivity to Temozolomide and low-dose ionizing radiation treatments in human glioblastoma cells. Cancer Biol Ther. 2013 May 10;14(7). [Epub ahead of print]

# Cong ZX, Wang HD, Zhou Y, Wang JW, Pan H, Zhang DD, Zhang L, Zhu L. Temozolomide and irradiation combined treatment-induced Nrf2 activation increases chemoradiation sensitivity in human glioblastoma cells. J Neurooncol. 2014 Jan;116(1):41-8.

# Costa B, Bendinelli S, Gabelloni P, Da Pozzo E, Daniele S, Scatena F, Vanacore R, Campiglia P, Bertamino A, Gomez-Monterrey I, Sorriento D, Del Giudice C, Iaccarino G, Novellino E, Martini C. Human Glioblastoma Multiforme: p53 Reactivation by a Novel MDM2 Inhibitor. PLoS One. 2013 Aug 19;8(8):e72281. doi: 10.1371/journal.pone.0072281.

# Craig EA, Spiegelman VS.Inhibition of coding region determinant binding protein sensitizes melanoma cells to chemotherapeutic agents. Pigment Cell Melanoma Res. 2012 Jan;25(1):83-7. doi: 10.1111/j.1755-148X.2011.00921.x. Epub 2011 Oct 28.

# Croucher JL1, Iyer R, Li N, Molteni V, Loren J, Gordon WP, Tuntland T, Liu B, Brodeur GM. TrkB inhibition by GNF-4256 slows growth and enhances chemotherapeutic efficacy in neuroblastoma xenografts. Cancer Chemother Pharmacol. 2015 Jan;75(1):131-41. doi: 10.1007/s00280-014-2627-1. Epub 2014 Nov 14.

# Dai C, Zhang B, Liu X, Ma S, Yang Y, Yao Y, Feng M, Bao X, Li G, Wang J, Guo K, Ma W, Xing B, Lian W, Xiao J, Cai F, Zhang H, Wang R. Inhibition of PI3K/AKT/mTOR pathway enhances temozolomide-induced cytotoxicity in pituitary adenoma cell lines in vitro and xenografted pituitary adenoma in female nude mice. Endocrinology. 2013 Mar;154(3):1247-59. doi: 10.1210/en.2012-1908. Epub 2013 Feb 5.

# Dittmann LM1, Danner A, Gronych J, Wolter M, Stühler K, Grzendowski M, Becker N, Bageritz J, Goidts V, Toedt G, Felsberg J, Sabel MC, Barbus S, Reifenberger G, Lichter P, Tews B. Downregulation of PRDX1 by promoter hypermethylation is frequent in 1p/19q-deleted oligodendroglial tumours and increases radio- and chemosensitivity of Hs683 glioma cells in vitro. Oncogene. 2012 Jul 19;31(29):3409-18. doi: 10.1038/onc.2011.513. Epub 2011 Dec 12.

# Dong F1, Eibach M1, Bartsch JW1, Dolga AM1, Schlomann U1, Conrad C1, Schieber S1, Schilling O1, Biniossek ML1, Culmsee C1, Strik H1, Koller G1, Carl B1, Nimsky C1. The metalloprotease-disintegrin ADAM8 contributes to temozolomide chemoresistance and enhanced invasiveness of human glioblastoma cells. Neuro Oncol. 2015 Mar 29. pii: nov042. [Epub ahead of print]

# Drucker KL, Paulsen AR, Giannini C, Decker PA, Blaber SI, Blaber M, Uhm JH, O'Neill BP, Jenkins RB, Scarisbrick IA. Clinical significance and novel mechanism of action of kallikrein 6 in glioblastoma. Neuro Oncol. 2013 Jan 10. [Epub ahead of print]

# Eich M, Roos WP, Dianov GL, Digweed M, Kaina B. Nijmegen breakage syndrome protein (NBN) causes resistance to methylating anticancer drugs such as temozolomide. Mol Pharmacol. 2010 Nov;78(5):943-51. doi: 10.1124/mol.110.066076. Epub 2010 Aug 20.

# Eich M, Roos WP, Nikolova T, Kaina B. Contribution of ATM and ATR to the resistance of glioblastoma and malignant melanoma cells to the methylating anticancer drug temozolomide. Mol Cancer Ther. 2013 Aug 19. [Epub ahead of print]

# Figul M1, Söling A, Dong HJ, Chou TC, Rainov NG. Combined effects of temozolomide and the ribonucleotide reductase inhibitors didox and trimidox in malignant brain tumor cells. Cancer Chemother Pharmacol. 2003 Jul;52(1):41-6. Epub 2003 Apr 11.

# Fiveash JB, Gillespie GY, Oliver PG, Zhou T, Belenky ML, Buchsbaum DJ. Enhancement of glioma radiotherapy and chemotherapy response with targeted antibody therapy against death receptor 5. Int J Radiat Oncol Biol Phys. 2008 Jun 1;71(2):507-16. doi: 10.1016/j.ijrobp.2008.02.005.

# Fontijn D, Adema AD, Bhakat KK, Pinedo HM, Peters GJ, Boven E.O6-methylguanine-DNA-methyltransferase promoter demethylation is involved in basic fibroblast growth factor induced resistance against temozolomide in human melanoma cells. Mol Cancer Ther. 2007 Oct;6(10):2807-15.

# G B1, Arfuso F2, Millward M3, Dharmarajan A4, Warrier S5. Secreted frizzled-related protein 4 inhibits glioma stem-like cells by reversing epithelial to mesenchymal transition, inducing apoptosis and decreasing cancer stem cell properties. PLoS One. 2015 Jun 1;10(6):e0127517. doi: 10.1371/journal.pone.0127517. eCollection 2015.

# Gao Y, Fotovati A, Lee C, Wang M, Cote G, Guns E, Toyota B, Faury D, Jabado N, Dunn SE.Inhibition of Y-box binding protein-1 slows the growth of glioblastoma multiforme and sensitizes to temozolomide independent O6-methylguanine-DNA methyltransferase. Mol Cancer Ther. 2009 Dec;8(12):3276-84. doi: 10.1158/1535-7163.MCT-09-0478.

# Geoerger B, Brasme JF, Daudigeos-Dubus E, Opolon P, Venot C, Debussche L, Vrignaud P, Vassal G. Anti-insulin-like growth factor 1 receptor antibody EM164 (murine AVE1642) exhibits anti-tumour activity alone and in combination with temozolomide against neuroblastoma. Eur J Cancer. 2010 Dec;46(18):3251-62. doi: 10.1016/j.ejca.2010.06.005. Epub 2010 Jun 28.

# Gielen PR, Aftab Q, Ma N, Chen VC, Hong X, Lozinsky S, Naus CC, Sin WC. Connexin43 confers Temozolomide resistance in human glioma cells by modulating the mitochondrial apoptosis pathway. Neuropharmacology. 2013 May 18. pii: S0028-3908(13)00211-6. doi: 10.1016/j.neuropharm.2013.05.002. [Epub ahead of print]

# Gimenez M1, Marie SK, Oba-Shinjo SM, Uno M, da Silva R, Laure HJ, Izumi C, Otake A, Chammas R, Rosa JC. Quantitative proteomic analysis and functional studies reveal that nucleophosmin is involved in cell death in glioblastoma cell line transfected with siRNA. Proteomics. 2012 Aug;12(17):2632-40. doi: 10.1002/pmic.201200034.

# Giussani P, Bassi R, Anelli V, Brioschi L, De Zen F, Riccitelli E, Caroli M, Campanella R, Gaini SM, Viani P, Riboni L. Glucosylceramide synthase protects glioblastoma cells against autophagic and apoptotic death induced by temozolomide and Paclitaxel. Cancer Invest. 2012 Jan;30(1):27-37. doi: 10.3109/07357907.2011.629379.

# Gratas C1, Séry Q, Rabé M, Oliver L, Vallette FM. Bak and Mcl-1 are essential for Temozolomide induced cell death in human glioma. Oncotarget. 2014 Jan 1. [Epub ahead of print]

# Haemmig S1, Baumgartner U1, Glück A1, Zbinden S1, Tschan MP1, Kappeler A1, Mariani L2, Vajtai I1, Vassella E1. miR-125b controls apoptosis and temozolomide resistance by targeting TNFAIP3 and NKIRAS2 in glioblastomas. Cell Death Dis. 2014 Jun 5;5:e1279. doi: 10.1038/cddis.2014.245.

# Happold C1, Roth P, Silginer M, Florea AM, Lamszus K, Frei K, Deenen R, Reifenberger G, Weller M. Interferon-β induces loss of spherogenicity and overcomes therapy resistance of glioblastoma stem cells. Mol Cancer Ther. 2014 Feb 13. [Epub ahead of print]

# Hattermann K, Mentlein R, Held-Feindt J. CXCL12 mediates apoptosis resistance in rat C6 glioma cells. Oncol Rep. 2012 May;27(5):1348-52. doi: 10.3892/or.2012.1674. Epub 2012 Feb 6.

# Hayashi T, Adachi K, Ohba S, Hirose Y. The Cdk inhibitor flavopiridol enhances temozolomide-induced cytotoxicity in human glioma cells. J Neurooncol. 2013 Aug 13. [Epub ahead of print]

# Held-Feindt J, Schmelz S, Hattermann K, Mentlein R, Mehdorn HM, Sebens S. The neural adhesion molecule L1CAM confers chemoresistance in human glioblastomas. Neurochem Int. 2012 Dec;61(7):1183-91. doi: 10.1016/j.neuint.2012.08.011. Epub 2012 Aug 28.

# Hiddingh L1, Raktoe RS2, Jeuken J3, Hulleman E4, Noske DP5, Kaspers GJ6, Vandertop WP7, Wesseling P8, Wurdinger T9. Identification of temozolomide resistance factors in glioblastoma via integrative miRNA/mRNA regulatory network analysis. Sci Rep. 2014 Jun 11;4:5260. doi: 10.1038/srep05260.

# Hiddingh L1, Tannous BA, Teng J, Tops B, Jeuken J, Hulleman E, Boots-Sprenger SH, Vandertop WP, Noske DP, Kaspers GJ, Wesseling P, Wurdinger T. EFEMP1 induces γ-secretase/Notch-mediated temozolomide resistance in glioblastoma. Oncotarget. 2013 Dec 7. [Epub ahead of print]

# Hirose Y, Berger MS, Pieper RO. Abrogation of the Chk1-mediated G(2) checkpoint pathway potentiates temozolomide-induced toxicity in a p53-independent manner in human glioblastoma cells. Cancer Res. 2001 Aug 1;61(15):5843-9.

# Hirose Y, Katayama M, Mirzoeva OK, Berger MS, Pieper RO. Akt activation suppresses Chk2-mediated, methylating agent-induced G2 arrest and protects from temozolomide-induced mitotic catastrophe and cellular senescence. Cancer Res. 2005 Jun 1;65(11):4861-9.

# Hirose Y, Katayama M, Stokoe D, Haas-Kogan DA, Berger MS, Pieper RO.

# Horton TM, Jenkins G, Pati D, Zhang L, Dolan ME, Ribes-Zamora A, Bertuch AA, Blaney SM, Delaney SL, Hegde M, Berg SL. Poly(ADP-ribose) polymerase inhibitor ABT-888 potentiates the cytotoxic activity of temozolomide in leukemia cells: influence of mismatch repair status and O6-methylguanine-DNA methyltransferase activity. Mol Cancer Ther. 2009 Aug;8(8):2232-42. doi: 10.1158/1535-7163.MCT-09-0142. Epub 2009 Aug 11.

# Huang H, Lin H, Zhang X, Li J. Resveratrol reverses temozolomide resistance by downregulation of MGMT in T98G glioblastoma cells by the NF-κB-dependent pathway. Oncol Rep. 2012 Jun;27(6):2050-6. doi: 10.3892/or.2012.1715. Epub 2012 Mar 12.

# Huang T, Jin X, He L, Zhang M, Wu J, Wang Y, Fang J. Role of podocalyxin in astrocytoma: Clinicopathological and in vitro evidence. Oncol Lett. 2013 Nov;6(5):1390-1396. Epub 2013 Sep 2.

# Huang ZM, Chinen M, Chang PJ, Xie T, Zhong L, Demetriou S, Patel MP, Scherzer R, Sviderskaya EV, Bennett DC, Millhauser GL, Oh DH, Cleaver JE, Wei ML. Targeting protein-trafficking pathways alters melanoma treatment sensitivity. Proc Natl Acad Sci U S A. 2012 Jan 10;109(2):553-8. doi: 10.1073/pnas.1118366109. Epub 2011 Dec 27.

# Jakubowicz-Gil J1, Langner E, Bądziul D, Wertel I, Rzeski W.Silencing of Hsp27 and Hsp72 in glioma cells as a tool for programmed cell death induction upon temozolomide and quercetin treatment. Toxicol Appl Pharmacol. 2013 Dec 15;273(3):580-9. doi: 10.1016/j.taap.2013.10.003. Epub 2013 Oct 12.

# Janouskova H, Maglott A, Leger DY, Bossert C, Noulet F, Guerin E, Guenot D, Pinel S, Chastagner P, Plenat F, Entz-Werle N, Lehmann-Che J, Godet J, Martin S, Teisinger J, Dontenwill M. Integrin α5β1 plays a critical role in resistance to temozolomide by interfering with the p53 pathway in high-grade glioma. Cancer Res. 2012 Jul 15;72(14):3463-70. doi: 10.1158/0008-5472.CAN-11-4199. Epub 2012 May 16.

# Javle M, Curtin NJ. The potential for poly (ADP-ribose) polymerase inhibitors in cancer therapy. Ther Adv Med Oncol. 2011 Nov;3(6):257-67. doi: 10.1177/1758834011417039.

# Johannessen TC, Prestegarden L, Grudic A, Hegi ME, Tysnes BB, Bjerkvig R. The DNA repair protein ALKBH2 mediates temozolomide resistance in human glioblastoma cells. Neuro Oncol. 2012 Dec 20. [Epub ahead of print]

# Keating AK, Kim GK, Jones AE, Donson AM, Ware K, Mulcahy JM, Salzberg DB, Foreman NK, Liang X, Thorburn A, Graham DK. Inhibition of Mer and Axl receptor tyrosine kinases in astrocytoma cells leads to increased apoptosis and improved chemosensitivity. Mol Cancer Ther. 2010 May;9(5):1298-307. doi: 10.1158/1535-7163.MCT-09-0707. Epub 2010 Apr 27.

# Kessler T1, Sahm F2,3, Blaes J1, Osswald M1,4, Rübmann P1, Milford D5, Urban S6, Jestaedt L5, Heiland S5, Bendszus M5, Hertenstein A7,4, Pfenning PN1, Ruiz de Almodóvar C6, Wick A4, Winkler F1,4, von Deimling A2,3, Platten M7,4, Wick W1,4, Weiler M1,4,8. Glioma cell VEGFR-2 confers resistance to chemotherapeutic and antiangiogenic treatments in PTEN-deficient glioblastoma. Oncotarget. 2015 Jan 13. [Epub ahead of print]

# Kim JW1, Kim JY1, Kim JE1, Kim SK2, Chung HT1, Park CK1.HOXA10 is associated with temozolomide resistance through regulation of the homologous recombinant DNA repair pathway in glioblastoma cell lines. Genes Cancer. 2014 May;5(5-6):165-74.

# Kitange GJ, Carlson BL, Schroeder MA, Decker PA, Morlan BW, Wu W, Ballman KV, Giannini C, Sarkaria JN.Expression of CD74 in high grade gliomas: a potential role in temozolomide resistance. J Neurooncol. 2010 Nov;100(2):177-86. doi: 10.1007/s11060-010-0186-9. Epub 2010 May 5.

# Kohsaka S, Wang L, Yachi K, Mahabir R, Narita T, Itoh T, Tanino M, Kimura T, Nishihara H, Tanaka S. STAT3 inhibition overcomes temozolomide resistance in glioblastoma by downregulating MGMT expression. Mol Cancer Ther. 2012 Jun;11(6):1289-99. doi: 10.1158/1535-7163.MCT-11-0801. Epub 2012 Apr 24.

# Kondo N, Takahashi A, Mori E, Noda T, Zdzienicka MZ, Thompson LH, Helleday T, Suzuki M, Kinashi Y, Masunaga S, Ono K, Hasegawa M, Ohnishi TFANCD1/BRCA2 plays predominant role in the repair of DNA damage induced by ACNU or TMZ. PLoS One. 2011 May 9;6(5):e19659. doi: 10.1371/journal.pone.0019659.

# Kondo N, Takahashi A, Mori E, Ohnishi K, McKinnon PJ, Sakaki T, Nakase H, Ohnishi T. DNA ligase IV as a new molecular target for temozolomide. Biochem Biophys Res Commun. 2009 Oct 2;387(4):656-60. doi: 10.1016/j.bbrc.2009.07.045. Epub 2009 Jul 15.

# Kreth S, Limbeck E, Hinske LC, Schütz SV, Thon N, Hoefig K, Egensperger R, Kreth FW. In human glioblastomas transcript elongation by alternative polyadenylation and miRNA targeting is a potent mechanism of MGMT silencing. Acta Neuropathol. 2013 Jan 23. [Epub ahead of print]

# Krzyzankova M, Mertsch S, Koos B, Jeibmann A, Kruse A, Kordes U, Frühwald MC, Wolff JE, Paulus W, Hasselblatt M. Loss of TP53 expression in immortalized choroid plexus epithelial cells results in increased resistance to anticancer agents. J Neurooncol. 2012 Sep;109(3):449-55. doi: 10.1007/s11060-012-0915-3. Epub 2012 Jul 5.

# Kumar DM, Patil V, Ramachandran B, Nila MV, Dharmalingam K, Somasundaram K. Temozolomide modulated glioma proteome: Role of Interleukin-1 receptor associated kinase-4 (IRAK4) in chemosensitivity. Proteomics. 2013 Apr 18. doi: 10.1002/pmic.201200261. [Epub ahead of print]

# Le Calvé B, Rynkowski M, Le Mercier M, Bruyère C, Lonez C, Gras T, Haibe-Kains B, Bontempi G, Decaestecker C, Ruysschaert JM, Kiss R, Lefranc F. Long-term in vitro treatment of human glioblastoma cells with temozolomide increases resistance in vivo through up-regulation of GLUT transporter and aldo-keto reductase enzyme AKR1C expression. Neoplasia. 2010 Sep;12(9):727-39.

# Le Mercier M, Lefranc F, Mijatovic T, Debeir O, Haibe-Kains B, Bontempi G, Decaestecker C, Kiss R, Mathieu V. Evidence of galectin-1 involvement in glioma chemoresistance. Toxicol Appl Pharmacol. 2008 Jun 1;229(2):172-83. doi: 10.1016/j.taap.2008.01.009. Epub 2008 Jan 29.

# Lee D1, Sun S1, Zhang XQ1, Zhang PD1, Ho AS1, Kiang KM1, Fung CF1, Lui WM1, Leung GK1. MicroRNA-210 and Endoplasmic Reticulum Chaperones in the Regulation of Chemoresistance in Glioblastoma. J Cancer. 2015 Jan 16;6(3):227-32. doi: 10.7150/jca.10765. eCollection 2015.

# Levati L, Ruffini F, Muzi A, Umezawa K, Graziani G, D'Atri S, Lacal PM. Placenta growth factor induces melanoma resistance to temozolomide through a mechanism that involves the activation of the transcription factor NF-κB. Int J Oncol. 2011 Jan;38(1):241-7.

# Li B, He H, Tao BB, Zhao ZY, Hu GH, Luo C, Chen JX, Ding XH, Sheng P, Dong Y, Zhang L, Lu YC. Knockdown of CDK6 enhances glioma sensitivity to chemotherapy. Oncol Rep. 2012 Sep;28(3):909-14. doi: 10.3892/or.2012.1884.

# Li L, Hu Y, Ylivinkka I, Li H, Chen P, Keski-Oja J, Hyytiäinen M. NETRIN-4 Protects Glioblastoma Cells from Temozolomide Induced Senescence. PLoS One. 2013 Nov 12;8(11):e80363. doi: 10.1371/journal.pone.0080363.

# Li RY, Chen LC, Zhang HY, Du WZ, Feng Y, Wang HB, Wen JQ, Liu X, Li XF, Sun Y, Yang DB, Jiang T, Li YL, Jiang CL. MiR-139 Inhibits Mcl-1 Expression and Potentiates TMZ-Induced Apoptosis in Glioma. CNS Neurosci Ther. 2013 Apr 2. doi: 10.1111/cns.12089. [Epub ahead of print]

# Liu G, Akasaki Y, Khong HT, Wheeler CJ, Das A, Black KL, Yu JS. Cytotoxic T cell targeting of TRP-2 sensitizes human malignant glioma to chemotherapy. Oncogene. 2005 Aug 4;24(33):5226-34.

# Liu X1, Ding Z2, Liu Y3, Zhang J1, Liu F2, Wang X3, He X2, Cui G4, Wang D5.Glycinamide ribonucleotide formyl transferase is frequently overexpressed in glioma and critically regulates the proliferation of glioma cells. Pathol Res Pract. 2013 Nov 15. pii: S0344-0338(13)00369-5. doi: 10.1016/j.prp.2013.10.009. [Epub ahead of print]

# Luo Y, Ellis LZ, Dallaglio K, Takeda M, Robinson WA, Robinson SE, Liu W, Lewis KD, McCarter MD, Gonzalez R, Norris DA, Roop DR, Spritz RA, Ahn NG, Fujita M.

# Ma J, Murphy M, O'Dwyer PJ, Berman E, Reed K, Gallo JM. Biochemical changes associated with a multidrug-resistant phenotype of a human glioma cell line with temozolomide-acquired resistance. Biochem Pharmacol. 2002 Apr 1;63(7):1219-28.

# Mansour NM1, Bernal GM1, Wu L1, Crawley CD1, Cahill KE1, Voce DJ1, Balyasnikova IV1, Zhang W2, Spretz R3, Nunez L3, Larsen GF3, Weichselbaum RR4, Yamini B5. Decoy Receptor DcR1 Is Induced in a p50/Bcl3-Dependent Manner and Attenuates the Efficacy of Temozolomide. Cancer Res. 2015 May 15;75(10):2039-48. doi: 10.1158/0008-5472.CAN-14-2144. Epub 2015 Mar 25.

# Martín V1, Sanchez-Sanchez AM, Herrera F, Gomez-Manzano C, Fueyo J, Alvarez-Vega MA, Antolín I, Rodriguez C. Melatonin-induced methylation of the ABCG2/BCRP promoter as a novel mechanism to overcome multidrug resistance in brain tumour stem cells. Br J Cancer. 2013 May 28;108(10):2005-12. doi: 10.1038/bjc.2013.188. Epub 2013 Apr 30.

# Martiniova L, Lu J, Chiang J, Bernardo M, Lonser R, Zhuang Z, Pacak K.Pharmacologic modulation of serine/threonine phosphorylation highly sensitizes PHEO in a MPC cell and mouse model to conventional chemotherapy. PLoS One. 2011 Feb 14;6(2):e14678. doi: 10.1371/journal.pone.0014678.

# Mathieu V, Le Mercier M, De Neve N, Sauvage S, Gras T, Roland I, Lefranc F, Kiss R. Galectin-1 knockdown increases sensitivity to temozolomide in a B16F10 mouse metastatic melanoma model. J Invest Dermatol. 2007 Oct;127(10):2399-410. Epub 2007 May 10.

# McNeill DR, Lam W, DeWeese TL, Cheng YC, Wilson DM 3rd. Impairment of APE1 function enhances cellular sensitivity to clinically relevant alkylators and antimetabolites. Mol Cancer Res. 2009 Jun;7(6):897-906. doi: 10.1158/1541-7786.MCR-08-0519. Epub 2009 May 26.

# Miyazaki T, Moritake K, Yamada K, Hara N, Osago H, Shibata T, Akiyama Y, Tsuchiya M. Indoleamine 2,3-dioxygenase as a new target for malignant glioma therapy. Laboratory investigation. J Neurosurg. 2009 Aug;111(2):230-7. doi: 10.3171/2008.10.JNS081141.

# Morales AG, Pezuk JA, Brassesco MS, de Oliveira JC, de Paula Queiroz RG, Machado HR, Carlotti CG Jr, Neder L, de Oliveira HF, Scrideli CA, Tone LG. BUB1 and BUBR1 inhibition decreases proliferation and colony formation, and enhances radiation sensitivity in pediatric glioblastoma cells. Childs Nerv Syst. 2013 Jun 2. [Epub ahead of print]

# Munoz JL1, Bliss SA, Greco SJ, Ramkissoon SH, Ligon KL, Rameshwar P. Delivery of Functional Anti-miR-9 by Mesenchymal Stem Cell-derived Exosomes to Glioblastoma Multiforme Cells Conferred Chemosensitivity. Mol Ther Nucleic Acids. 2013 Oct 1;2:e126. doi: 10.1038/mtna.2013.60.

# Sun S, Lee D, Ho AS, Pu JK, Zhang XQ, Lee NP, Day PJ, Lui WM, Fung CF, Leung GK. Inhibition of prolyl 4-hydroxylase, beta polypeptide (P4HB) attenuates temozolomide resistance in malignant glioma via the endoplasmic reticulum stress response (ERSR) pathways. Neuro Oncol. 2013 May;15(5):562-77. doi: 10.1093/neuonc/not005. Epub 2013 Feb 26.

# Niibori-Nambu A, Midorikawa U, Mizuguchi S, Hide T, Nagai M, Komohara Y, Nagayama M, Hirayama M, Kobayashi D, Tsubota N, Takezaki T, Makino K, Nakamura H, Takeya M, Kuratsu J, Araki N. Glioma Initiating Cells Form a Differentiation Niche Via the Induction of Extracellular Matrices and Integrin αV. PLoS One. 2013 May 21;8(5):e59558. doi: 10.1371/journal.pone.0059558. Print 2013.

# Okada M1, Sato A1, Shibuya K1, Watanabe E1, Seino S1, Suzuki S1, Seino M1, Narita Y2, Shibui S2, Kayama T3, Kitanaka C1. JNK contributes to temozolomide resistance of stem-like glioblastoma cells via regulation of MGMT expression. Int J Oncol. 2014 Feb;44(2):591-9. doi: 10.3892/ijo.2013.2209. Epub 2013 Dec 5.

# Oliva CR, Nozell SE, Diers A, McClugage SG 3rd, Sarkaria JN, Markert JM, Darley-Usmar VM, Bailey SM, Gillespie GY, Landar A, Griguer CE. Acquisition of temozolomide chemoresistance in gliomas leads to remodeling of mitochondrial electron transport chain. J Biol Chem. 2010 Dec 17;285(51):39759-67. doi: 10.1074/jbc.M110.147504. Epub 2010 Sep 24.

# Pan SJ1, Wu YB2, Cai S3, Pan YX4, Liu W4, Bian LG1, Sun B4, Sun QF5.Over-expression of tetraspanin 8 in malignant glioma regulates tumor cell progression. Biochem Biophys Res Commun. 2015 Mar 13;458(3):476-82. doi: 10.1016/j.bbrc.2015.01.128. Epub 2015 Feb 11.

# Passagne I, Evrard A, Depeille P, Cuq P, Cupissol D, Vian L. O(6)-methylguanine DNA-methyltransferase (MGMT) overexpression in melanoma cells induces resistance to nitrosoureas and temozolomide but sensitizes to mitomycin C. Toxicol Appl Pharmacol. 2006 Mar 1;211(2):97-105. Epub 2005 Jul 22.

# Peigñan L, Garrido W, Segura R, Melo R, Rojas D, Cárcamo JG, San Martín R, Quezada C. Combined use of anticancer drugs and an inhibitor of multiple drug resistance-associated protein-1 increases sensitivity and decreases survival of glioblastoma multiforme cells in vitro. Neurochem Res. 2011 Aug;36(8):1397-406. doi: 10.1007/s11064-011-0464-8. Epub 2011 May 5.

# Persano L, Pistollato F, Rampazzo E, Della Puppa A, Abbadi S, Frasson C, Volpin F, Indraccolo S, Scienza R, Basso G. BMP2 sensitizes glioblastoma stem-like cells to Temozolomide by affecting HIF-1α stability and MGMT expression. Cell Death Dis. 2012 Oct 18;3:e412. doi: 10.1038/cddis.2012.153.

# Pezuk JA, Brassesco MS, Morales AG, de Oliveira JC, de Paula Queiroz RG, Machado HR, Carlotti CG Jr, Neder L, Scrideli CA, Tone LG. Polo-like kinase 1 inhibition causes decreased proliferation by cell cycle arrest, leading to cell death in glioblastoma. Cancer Gene Ther. 2013 Jul 26. doi: 10.1038/cgt.2013.46. [Epub ahead of print]

# Pojo M1,2, Gonçalves CS1,2, Xavier-Magalhães A1,2, Oliveira AI1,2, Gonçalves T1,2, Correia S3, Rodrigues AJ1,2, Costa S1,2, Pinto L1,2, Pinto AA4, Lopes JM5,6,7, Reis RM1,2,8, Rocha M3, Sousa N1,2, Costa BM1,2. A transcriptomic signature mediated by HOXA9 promotes human glioblastoma initiation, aggressiveness and resistance to temozolomide. Oncotarget. 2015 Apr 10;6(10):7657-74.

# Pyko IV, Nakada M, Sabit H, Lei T, Furuyama N, Hayashi Y, Kawakami K, Minamoto T, Fedulau AS, Hamada J. Glycogen synthase kinase 3β inhibition sensitizes human glioblastoma cells to temozolomide by affecting O6-methylguanine DNA methyltransferase promoter methylation via c-Myc signaling. Carcinogenesis. 2013 May 28. [Epub ahead of print]

# Pyrko P, Schönthal AH, Hofman FM, Chen TC, Lee AS. The unfolded protein response regulator GRP78/BiP as a novel target for increasing chemosensitivity in malignant gliomas. Cancer Res. 2007 Oct 15;67(20):9809-16.

# Qi XC, Xie DJ, Yan QF, Wang YR, Zhu YX, Qian C, Yang SX. LRIG1 Dictates the Chemo-sensitivity of Temozolomide (TMZ) in U251 Glioblastoma Cells via Down-regulation of EGFR/Topoisomerase-2/Bcl-2. Biochem Biophys Res Commun. 2013 Jul 9. pii: S0006-291X(13)01126-1. doi: 10.1016/j.bbrc.2013.06.116. [Epub ahead of print]

# Quann K, Gonzales DM, Mercier I, Wang C, Sotgia F, Pestell RG, Lisanti MP, Jasmin JF. Caveolin-1 is a negative regulator of tumor growth in glioblastoma and modulates chemosensitivity to temozolomide. Cell Cycle. 2013 Apr 17;12(10). [Epub ahead of print]

# Quiros S1, Roos WP, Kaina B. Rad51 and BRCA2--New molecular targets for sensitizing glioma cells to alkylating anticancer drugs. PLoS One. 2011;6(11):e27183. doi: 10.1371/journal.pone.0027183. Epub 2011 Nov 2.

# Riccitelli E, Giussani P, Di Vito C, Condomitti G, Tringali C, Caroli M, Galli R, Viani P, Riboni L.Extracellular sphingosine-1-phosphate: a novel actor in human glioblastoma stem cell survival. PLoS One. 2013 Jun 24;8(6):e68229. doi: 10.1371/journal.pone.0068229. Print 2013.

# Riganti C, Salaroglio IC, Caldera V, Campia I, Kopecka J, Mellai M, Annovazzi L, Bosia A, Ghigo D, Schiffer D. Temozolomide downregulates P-glycoprotein expression in glioblastoma stem cells by interfering with the Wnt3a/glycogen synthase-3 kinase/β-catenin pathway. Neuro Oncol. 2013 Jul 28. [Epub ahead of print]

# Roos WP, Jöst E, Belohlavek C, Nagel G, Fritz G, Kaina B. Intrinsic anticancer drug resistance of malignant melanoma cells is abrogated by IFN-β and valproic acid. Cancer Res. 2011 Jun 15;71(12):4150-60. doi: 10.1158/0008-5472.CAN-10-3498. Epub 2011 Apr 14.

# Roos WP, Tsaalbi-Shtylik A, Tsaryk R, Güvercin F, de Wind N, Kaina B. The translesion polymerase Rev3L in the tolerance of alkylating anticancer drugs. Mol Pharmacol. 2009 Oct;76(4):927-34. doi: 10.1124/mol.109.058131. Epub 2009 Jul 29.

# Rosati SF, Williams RF, Nunnally LC, McGee MC, Sims TL, Tracey L, Zhou J, Fan M, Ng CY, Nathwani AC, Stewart CF, Pfeffer LM, Davidoff AM. IFN-beta sensitizes neuroblastoma to the antitumor activity of temozolomide by modulating O6-methylguanine DNA methyltransferase expression. Mol Cancer Ther. 2008 Dec;7(12):3852-8. doi: 10.1158/1535-7163.MCT-08-0806. Epub 2008 Dec 3.

# Sang DP1, Li RJ2, Lan Q2. Quercetin sensitizes human glioblastoma cells to temozolomide in vitro via inhibition of Hsp27. Acta Pharmacol Sin. 2014 Jun;35(6):832-8. doi: 10.1038/aps.2014.22.

# Sato A, Sunayama J, Matsuda K, Seino S, Suzuki K, Watanabe E, Tachibana K, Tomiyama A, Kayama T, Kitanaka C. MEK-ERK signaling dictates DNA-repair gene MGMT expression and temozolomide resistance of stem-like glioblastoma cells via the MDM2-p53 axis. Stem Cells. 2011 Dec;29(12):1942-51. doi: 10.1002/stem.753.

# Schäfer A, Teufel J, Ringel F, Bettstetter M, Hoepner I, Rasper M, Gempt J, Koeritzer J, Schmidt-Graf F, Meyer B, Beier CP, Schlegel J. Aldehyde dehydrogenase 1A1--a new mediator of resistance to temozolomide in glioblastoma. Neuro Oncol. 2012 Nov 6. [Epub ahead of print]

# She X1, Yu Z, Cui Y, Lei Q, Wang Z, Xu G, Luo Z, Li G, Wu M. miR-181 subunits enhance the chemosensitivity of temozolomide by Rap1B-mediated cytoskeleton remodeling in glioblastoma cells. Med Oncol. 2014 Apr;31(4):892. doi: 10.1007/s12032-014-0892-9. Epub 2014 Feb 27.

# Shervington A, Patel R.Silencing DNA methyltransferase (DNMT) enhances glioma chemosensitivity. Oligonucleotides. 2008 Dec;18(4):365-74. doi: 10.1089/oli.2008.0128.

# Shi L, Zhang S, Feng K, Wu F, Wan Y, Wang Z, Zhang J, Wang Y, Yan W, Fu Z, You Y. MicroRNA-125b-2 confers human glioblastoma stem cells resistance to temozolomide through the mitochondrial pathway of apoptosis. Int J Oncol. 2012 Jan;40(1):119-29. doi: 10.3892/ijo.2011.1179. Epub 2011 Aug 29.

# Shi Z, Chen Q, Li C, Wang L, Qian X, Jiang C, Liu X, Wang X, Li H, Kang C, Jiang T, Liu LZ, You Y, Liu N, Jiang BH. MiR-124 governs glioma growth and angiogenesis and enhances chemosensitivity by targeting R-Ras and N-Ras. Neuro Oncol. 2014 May 25. pii: nou084. [Epub ahead of print]

# Shinsato Y, Furukawa T, Yunoue S, Yonezawa H, Minami K, Nishizawa Y, Ikeda R, Kawahara K, Yamamoto M, Hirano H, Tokimura H, Arita K. Reduction of MLH1 and PMS2 confers temozolomide resistance and is associated with recurrence of glioblastoma. Oncotarget. 2013 Oct 14. [Epub ahead of print]

# Shukla S1, Patric IR1, Patil V1, Shwetha SD2, Hegde AS3, Chandramouli BA2, Arivazhagan A2, Santosh V2, Somasundaram K4. Methylation Silencing of ULK2, An Autophagy Gene, is Essential for Astrocyte Transformation and Tumor Growth. J Biol Chem. 2014 Jun 12. pii: jbc.M114.567032. [Epub ahead of print]

# Side population cells from human melanoma tumors reveal diverse mechanisms for chemoresistance. J Invest Dermatol. 2012 Oct;132(10):2440-50. doi: 10.1038/jid.2012.161. Epub 2012 May 24.

# Siebzehnrubl FA, Silver DJ, Tugertimur B, Deleyrolle LP, Siebzehnrubl D, Sarkisian MR, Devers KG, Yachnis AT, Kupper MD, Neal D, Nabilsi NH, Kladde MP, Suslov O, Brabletz S, Brabletz T, Reynolds BA, Steindler DA. The ZEB1 pathway links glioblastoma initiation, invasion and chemoresistance. EMBO Mol Med. 2013 Jul 1. doi: 10.1002/emmm.201302827. [Epub ahead of print]

# Silber JR, Bobola MS, Blank A, Schoeler KD, Haroldson PD, Huynh MB, Kolstoe DD. The apurinic/apyrimidinic endonuclease activity of Ape1/Ref-1 contributes to human glioma cell resistance to alkylating agents and is elevated by oxidative stress. Clin Cancer Res. 2002 Sep;8(9):3008-18.

# Srivenugopal KS, Shou J, Mullapudi SR, Lang FF Jr, Rao JS, Ali-Osman F. Enforced expression of wild-type p53 curtails the transcription of the O(6)-methylguanine-DNA methyltransferase gene in human tumor cells and enhances their sensitivity to alkylating agents. Clin Cancer Res. 2001 May;7(5):1398-409.

# Stevens BM1, Folts CJ, Cui W, Bardin AL, Walter K, Carson-Walter E, Vescovi A, Noble M. Cool-1 mediated inhibition of c-Cbl modulates multiple critical properties of glioblastomas, including the ability to generate tumors in vivo. Stem Cells. 2014 Jan 24. doi: 10.1002/stem.1644. [Epub ahead of print]

# Szeliga M, Zgrzywa A, Obara-Michlewska M, Albrecht J. Transfection of a human glioblastoma cell line with liver-type glutaminase (LGA) down-regulates the expression of DNA-repair gene MGMT and sensitizes the cells to alkylating agents. J Neurochem. 2012 Nov;123(3):428-36. doi: 10.1111/j.1471-4159.2012.07917.x. Epub 2012 Sep 21.

# Tang JB, Svilar D, Trivedi RN, Wang XH, Goellner EM, Moore B, Hamilton RL, Banze LA, Brown AR, Sobol RW. N-methylpurine DNA glycosylase and DNA polymerase beta modulate BER inhibitor potentiation of glioma cells to temozolomide. Neuro Oncol. 2011 May;13(5):471-86. doi: 10.1093/neuonc/nor011. Epub 2011 Mar 3.

# Tentori L, Muzi A, Dorio AS, Bultrini S, Mazzon E, Lacal PM, Shah GM, Zhang J, Navarra P, Nocentini G, Cuzzocrea S, Graziani G. Stable depletion of poly (ADP-ribose) polymerase-1 reduces in vivo melanoma growth and increases chemosensitivity. Eur J Cancer. 2008 Jun;44(9):1302-14. doi: 10.1016/j.ejca.2008.03.019. Epub 2008 Apr 24.

# The p38 mitogen-activated protein kinase pathway links the DNA mismatch repair system to the G2 checkpoint and to resistance to chemotherapeutic DNA-methylating agents. Mol Cell Biol. 2003 Nov;23(22):8306-15.

# Tian T1, Li A2, Lu H3, Luo R4, Zhang M2, Li Z5. TAZ promotes temozolomide resistance by upregulating MCL-1 in human glioma cells. Biochem Biophys Res Commun. 2015 Jun 1. pii: S0006-291X(15)30039-5. doi: 10.1016/j.bbrc.2015.05.115. [Epub ahead of print]

# Trivedi RN, Wang XH, Jelezcova E, Goellner EM, Tang JB, Sobol RW. Human methyl purine DNA glycosylase and DNA polymerase beta expression collectively predict sensitivity to temozolomide. Mol Pharmacol. 2008 Aug;74(2):505-16. doi: 10.1124/mol.108.045112. Epub 2008 May 13.

# Tsaryk R, Fabian K, Thacker J, Kaina B.Xrcc2 deficiency sensitizes cells to apoptosis by MNNG and the alkylating anticancer drugs temozolomide, fotemustine and mafosfamide. Cancer Lett. 2006 Aug 8;239(2):305-13. Epub 2005 Nov 18.

# Turner KM1, Sun Y1, Ji P1, Granberg KJ2, Bernard B3, Hu L1, Cogdell DE1, Zhou X1, Yli-Harja O4, Nykter M4, Shmulevich I3, Yung WK5, Fuller GN1, Zhang W6. Genomically amplified Akt3 activates DNA repair pathway and promotes glioma progression. Proc Natl Acad Sci U S A. 2015 Mar 17;112(11):3421-6. doi: 10.1073/pnas.1414573112. Epub 2015 Mar 3.

# Ujifuku K, Mitsutake N, Takakura S, Matsuse M, Saenko V, Suzuki K, Hayashi K, Matsuo T, Kamada K, Nagata I, Yamashita S. miR-195, miR-455-3p and miR-10a( *) are implicated in acquired temozolomide resistance in glioblastoma multiforme cells. Cancer Lett. 2010 Oct 28;296(2):241-8. doi: 10.1016/j.canlet.2010.04.013. Epub 2010 May 4.

# Ulasov I, Thaci B, Sarvaiya P, Yi R, Guo D, Auffinger B, Pytel P, Zhang L, Kim CK, Borovjagin A, Dey M, Han Y, Baryshnikov AY, Lesniak MS. Inhibition of MMP14 potentiates the therapeutic effect of temozolomide and radiation in gliomas. Cancer Med. 2013 Aug;2(4):457-67. doi: 10.1002/cam4.104. Epub 2013 Jun 30.

# Virrey JJ, Guan S, Li W, Schönthal AH, Chen TC, Hofman FM. Increased survivin expression confers chemoresistance to tumor-associated endothelial cells. Am J Pathol. 2008 Aug;173(2):575-85. doi: 10.2353/ajpath.2008.071079. Epub 2008 Jul 3.

# Voss V, Senft C, Lang V, Ronellenfitsch MW, Steinbach JP, Seifert V, Kögel D. The pan-Bcl-2 inhibitor (-)-gossypol triggers autophagic cell death in malignant glioma. Mol Cancer Res. 2010 Jul;8(7):1002-16. doi: 10.1158/1541-7786.MCR-09-0562. Epub 2010 Jun 29.

# Wan Y, Sun G, Zhang S, Wang Z, Shi L. MicroRNA-125b inhibitor sensitizes human primary glioblastoma cells to chemotherapeutic drug temozolomide on invasion. In Vitro Cell Dev Biol Anim. 2013 Jul 9. [Epub ahead of print]

# Wang J1, Sai K, Chen FR, Chen ZP. miR-181b modulates glioma cell sensitivity to temozolomide by targeting MEK1. Cancer Chemother Pharmacol. 2013 Jul;72(1):147-58. doi: 10.1007/s00280-013-2180-3. Epub 2013 May 5.

# Wang JB1, Dong DF, Wang MD, Gao K. IDH1 Overexpression Induced Chemotherapy Resistance and IDH1 Mutation Enhanced Chemotherapy Sensitivity in Glioma Cells in Vitro and in Vivo. Asian Pac J Cancer Prev. 2014;15(1):427-32.

# Wang Q, Qian J, Wang J, Luo C, Chen J, Hu G, Lu Y. Knockdown of RLIP76 expression by RNA interference inhibits invasion, induces cell cycle arrest, and increases chemosensitivity to the anticancer drug temozolomide in glioma cells. J Neurooncol. 2013 Mar;112(1):73-82. doi: 10.1007/s11060-013-1045-2. Epub 2013 Jan 6.

# Wang X, Chen JX, Liu YH, You C, Mao Q. Mutant TP53 enhances the resistance of glioblastoma cells to temozolomide by up-regulating O(6)-methylguanine DNA-methyltransferase. Neurol Sci. 2012 Dec 8. [Epub ahead of print]

# Wang Z1,2, Yang J2, Xu G2, Wang W2, Liu C2, Yang H2, Yu Z2, Lei Q2, Xiao L2, Xiong J2,3, Zeng L1, Xiang J1,2, Ma J1,2, Li G1,2, Wu M1,2. Targeting miR-381-NEFL axis sensitizes glioblastoma cells to temozolomide by regulating stemness factors and multidrug resistance factors. Oncotarget. 2015 Feb 20;6(5):3147-64.

# Weiler M1, Blaes J, Pusch S, Sahm F, Czabanka M, Luger S, Bunse L, Solecki G, Eichwald V, Jugold M, Hodecker S, Osswald M, Meisner C, Hielscher T, Rübmann P, Pfenning PN, Ronellenfitsch M, Kempf T, Schnölzer M, Abdollahi A, Lang F, Bendszus M, von Deimling A, Winkler F, Weller M, Vajkoczy P, Platten M, Wick W. mTOR target NDRG1 confers MGMT-dependent resistance to alkylating chemotherapy. Proc Natl Acad Sci U S A. 2014 Jan 7;111(1):409-14. doi: 10.1073/pnas.1314469111. Epub 2013 Dec 23.

# Wen X1, Huang A2, Liu Z1, Liu Y3, Hu J1, Liu J1, Shuai X4.Downregulation of ROCK2 through Nanocomplex Sensitizes the Cytotoxic Effect of Temozolomide in U251 Glioma Cells. PLoS One. 2014 Mar 18;9(3):e92050. doi: 10.1371/journal.pone.0092050. eCollection 2014.

# Wolf A, Agnihotri S, Micallef J, Mukherjee J, Sabha N, Cairns R, Hawkins C, Guha A. Hexokinase 2 is a key mediator of aerobic glycolysis and promotes tumor growth in human glioblastoma multiforme. J Exp Med. 2011 Feb 14;208(2):313-26. doi: 10.1084/jem.20101470. Epub 2011 Jan 17.

# Wong ST, Zhang XQ, Zhuang JT, Chan HL, Li CH, Leung GK. MicroRNA-21 inhibition enhances in vitro chemosensitivity of temozolomide-resistant glioblastoma cells. Anticancer Res. 2012 Jul;32(7):2835-41.

# Wu H, Liu Q, Cai T, Chen YD, Liao F, Wang ZF1. MiR-136 modulates glioma cell sensitivity to temozolomide by targeting astrocyte elevated gene-1. Diagn Pathol. 2014 Sep 30;9:173. doi: 10.1186/s13000-014-0173-0.

# Wu H, Yang L, Liao D, Chen Y, Wang W, Fang J. Podocalyxin regulates astrocytoma cell invasion and survival against temozolomide. Exp Ther Med. 2013 Apr;5(4):1025-1029. Epub 2013 Feb 15.

# Wu L1, Yang L, Xiong Y, Guo H, Shen X, Cheng Z, Zhang Y, Gao Z, Zhu X. Annexin A5 promotes invasion and chemoresistance to temozolomide in glioblastoma multiforme cells. Tumour Biol. 2014 Dec;35(12):12327-37. doi: 10.1007/s13277-014-2545-1. Epub 2014 Sep 23.

# Wu ZB, Cai L, Lin SJ, Xiong ZK, Lu JL, Mao Y, Yao Y, Zhou LF. High-mobility group box 2 is associated with prognosis of glioblastoma by promoting cell viability, invasion, and chemotherapeutic resistance. Neuro Oncol. 2013 Jul 4. [Epub ahead of print]

# Xie L, Meyskens FL Jr. The pan-Aurora kinase inhibitor, PHA-739358, induces apoptosis and inhibits migration in melanoma cell lines. Melanoma Res. 2013 Apr;23(2):102-13. doi: 10.1097/CMR.0b013e32835df5e4.

# Yan F1, Alinari L, Lustberg ME, Martin LK, Cordero-Nieves HM, Banasavadi-Siddegowda Y, Virk S, Barnholtz-Sloan J, Bell EH, Wojton J, Jacob NK, Chakravarti A, Nowicki MO, Wu X, Lapalombella R, Datta J, Yu B, Gordon K, Haseley A, Patton JT, Smith PL, Ryu J, Zhang X, Mo X, Marcucci G, Nuovo G, Kwon CH, Byrd JC, Chiocca EA, Li C, Sif S, Jacob S, Lawler S, Kaur B, Baiocchi RA. Genetic validation of the protein arginine methyltransferase PRMT5 as a candidate therapeutic target in glioblastoma. Cancer Res. 2014 Jan 22. [Epub ahead of print]

# Yeom SY1, Nam DH2, Park C3. RRAD promotes EGFR-mediated STAT3 activation and induces temozolomide resistance of malignant glioblastoma. Mol Cancer Ther. 2014 Dec;13(12):3049-61. doi: 10.1158/1535-7163.MCT-14-0244. Epub 2014 Oct 13.

# Yin D, Chen W, O'Kelly J, Lu D, Ham M, Doan NB, Xie D, Wang C, Vadgama J, Said JW, Black KL, Koeffler HP. Connective tissue growth factor associated with oncogenic activities and drug resistance in glioblastoma multiforme. Int J Cancer. 2010 Nov 15;127(10):2257-67. doi: 10.1002/ijc.25257.

# Yip S, Miao J, Cahill DP, Iafrate AJ, Aldape K, Nutt CL, Louis DN.MSH6 mutations arise in glioblastomas during temozolomide therapy and mediate temozolomide resistance. Clin Cancer Res. 2009 Jul 15;15(14):4622-9. doi: 10.1158/1078-0432.CCR-08-3012. Epub 2009 Jul 7.

# Yu H, Park J, Lee J, Choi K, Choi C. Constitutive Expression of MAP Kinase Phosphatase-1 Confers Multi-drug Resistance in Human Glioblastoma Cells. Cancer Res Treat. 2012 Sep;44(3):195-201. doi: 10.4143/crt.2012.44.3.195. Epub 2012 Sep 30.

# Zeng L1, Kang C2, Di C2, Fee BE3, Rivas M3, Lin J2, Adamson DC3. The adherens junction-associated protein 1 is a negative transcriptional regulator of MAGEA2, which potentiates temozolomide-induced apoptosis in GBM. Int J Oncol. 2014 Apr;44(4):1243-51. doi: 10.3892/ijo.2014.2277. Epub 2014 Jan 24.

# Zhang LH, Yin AA, Cheng JX, Huang HY, Li XM, Zhang YQ, Han N, Zhang X. TRIM24 promotes glioma progression and enhances chemoresistance through activation of the PI3K/Akt signaling pathway. Oncogene. 2014 Jan 27. doi: 10.1038/onc.2013.593. [Epub ahead of print]

# Zhang N, Wu X, Yang L, Xiao F, Zhang H, Zhou A, Huang Z, Huang S. FoxM1 Inhibition Sensitizes Resistant Glioblastoma Cells to Temozolomide by Downregulating the Expression of DNA Repair Gene Rad51. Clin Cancer Res. 2012 Sep 12. [Epub ahead of print]

# Zhang S, Wan Y, Pan T, Gu X, Qian C, Sun G, Sun L, Xiang Y, Wang Z, Shi L. MicroRNA-21 inhibitor sensitizes human glioblastoma U251 stem cells to chemotherapeutic drug temozolomide. J Mol Neurosci. 2012 Jun;47(2):346-56. doi: 10.1007/s12031-012-9759-8. Epub 2012 Apr 19.

# Zheng M, Bocangel D, Ramesh R, Ekmekcioglu S, Poindexter N, Grimm EA, Chada S.Interleukin-24 overcomes temozolomide resistance and enhances cell death by down-regulation of O6-methylguanine-DNA methyltransferase in human melanoma cells. Mol Cancer Ther. 2008 Dec;7(12):3842-51. doi: 10.1158/1535-7163.MCT-08-0516. Epub 2008 Dec 3.

# Zhou Y, Wang HD, Zhu L, Cong ZX, Li N, Ji XJ, Pan H, Wang JW, Li WC. Knockdown of Nrf2 enhances autophagy induced by temozolomide in U251 human glioma cell line. Oncol Rep. 2013 Jan;29(1):394-400. doi: 10.3892/or.2012.2115. Epub 2012 Oct 31.

# Zhou Y, Wang HD, Zhu L, Cong ZX, Li N, Ji XJ, Pan H, Wang JW, Li WC. Knockdown of Nrf2 enhances autophagy induced by temozolomide in U251 human glioma cell line. Oncol Rep. 2013 Jan;29(1):394-400. doi: 10.3892/or.2012.2115. Epub 2012 Oct 31.

# Zhuang D, Liu Y, Mao Y, Gao L, Zhang H, Luan S, Huang F, Li Q. TMZ-induced PrPc/par-4 interaction promotes the survival of human glioma cells. Int J Cancer. 2012 Jan 15;130(2):309-18. doi: 10.1002/ijc.25985. Epub 2011 May 30.

# Ziegler DS, Keating J, Kesari S, Fast EM, Zawel L, Ramakrishna N, Barnes J, Kieran MW, Veldhuijzen van Zanten SE, Kung AL. A small-molecule IAP inhibitor overcomes resistance to cytotoxic therapies in malignant gliomas in vitro and in vivo. Neuro Oncol. 2011 Aug;13(8):820-9. doi: 10.1093/neuonc/nor066. Epub 2011 Jul 1.
