# Supplementary figures and images for: Temozolomide promotes genomic and phenotypic changes in glioblastoma cells
Source: Cancer Cell Int. 2016 May 5;16:36. doi: 10.1186/s12935-016-0311-8 (PMC4858898; doi:10.1186/s12935-016-0311-8)

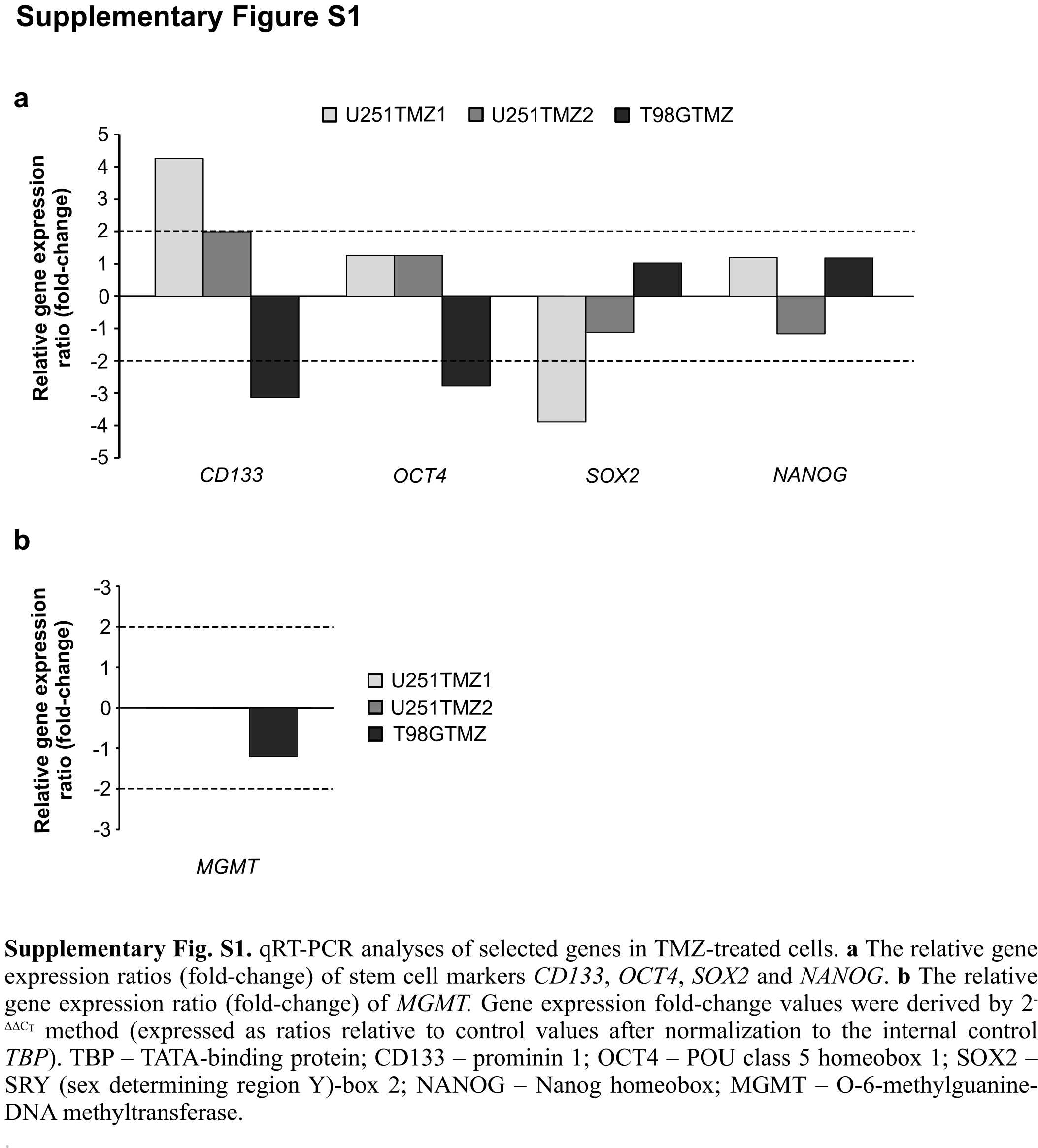

Supplement: Supplementary file 9 — 10.1186/s12935-016-0311-8qRT-PCR analysis of selected genes in TMZ-treated cells. a The relative gene expression ratios (fold-change) of stem cell markers CD133, OCT4, SOX2, and NANOG. b The relative gene expression ratio (fold-change) of MGMT. Gene expression fold-change values were derived by 2-ΔΔCT method (expressed as ratios relative to control values after normalization to the internal control TBP). TBP – TATA-binding protein; CD133 – prominin 1; OCT4 – POU class 5 homeobox 1; SOX2 – SRY (sex determining region Y)-box 2; NANOG – Nanog homeobox; MGMT – O-6-methylguanine-DNA methyltransferase. [file 12935_2016_311_MOESM9_ESM.tif]
